# Clinical features and current management experience in Gorham-Stout disease: a systematic review

**DOI:** 10.1186/s13023-025-03649-9

**Published:** 2025-03-19

**Authors:** Zilong Zhou, Tong Qiu, Jiangyuan Zhou, Zixin Zhang, Xue Gong, Xuepeng Zhang, Yuru Lan, Congxia Yang, Yujia Zhang, Shanshan Xiang, Yi Ji

**Affiliations:** 1 Division of Oncology, Department of Pediatric Surgery, West China Hospital, Sichuan University, Chengdu, Sichuan China; 2 Pediatric Intensive Care Unit, Department of Critical Care Medicine, West China Hospital, Sichuan University, Chengdu, Sichuan China

**Keywords:** Gorham-Stout disease, Epidemiology, Management, Pleural effusion, Differential diagnosis

## Abstract

**Background:**

Gorham-Stout disease (GSD) is a rare complex lymphatic malformation. Since its initial description in 1838, only approximately 400 patients have been documented. There is currently no consensus on the diagnostic criteria or treatment options for GSD. The objective of this study was to review the clinical characteristics of patients with GSD and determine the current diagnostic and treatment models.

**Methods:**

A comprehensive search of the PubMed, Web of Science, Embase, and Cochrane Library databases was conducted to identify all relevant literature on GSD published over the decade from 2013 to 2023. The clinical information extracted from these publications was analyzed.

**Results:**

A total of 206 patients with GSD were included in the study, comprising 119 males, 81 females and 6 patients with unknown sex. The age of onset of patients was widely distributed, ranging from 0 to 77 years old. However, the majority of cases occurred in childhood (50.7%). Fifteen patients (10.3%) exhibited an onset age of less than 1 year. The average time from the onset of symptoms to diagnosis was 3.5 years. The number of patients with osteolysis in the axial bone was greater than that in the appendiceal bone (*P* < 0.05), and the number of patients with multiple osteolytic lesions was greater than that with single osteolytic lesions (77.2% vs. 22.8%). In general, GSD was more likely to occur in the spine (46.1%), ribs (28.6%), hip (23.3%), femur (18.4%), mandible (15.5%) and humerus (15.0%). Pain was the most common symptom, with 68.4% of patients reporting pain in the lesion area. Surgery (66.9%) and bisphosphonates (56.9%) are still the mainstream treatment methods, with a total of 33 (18.2%) patients receiving sirolimus. Pleural effusion was identified as a risk factor for patient mortality (*P* < 0.05).

**Conclusions:**

GSD is most commonly observed in children, with a slight male predisposition. It commonly manifests as multiple osteolysis of the axial bone, with pain being the most common symptom. The presence of pleural effusion indicates a serious condition that requires close monitoring to prevent mortality. Despite the advent of novel therapeutic modalities, the management of GSD remains an area in need of further investigation.

**Supplementary Information:**

The online version contains supplementary material available at 10.1186/s13023-025-03649-9.

## Introduction

Gorham-Stout disease (GSD) is a rare lymphatic malformation of unknown etiology, and it is also known as vanishing bone disease, disappearing bone disease or massive osteolysis [1]. According to the classification by the International Society for the Study of Vascular Anomalies (ISSVA) updated in 2018, GSD is a subtype of lymphatic malformation [2]. In 1838, Jackson first reported a boy with humeral osteolysis [3], and subsequently, Gorham and Stout further clarified and defined this syndrome through multiple cases in 1955 [4]. Currently, nearly 400 GSD patients have been reported worldwide. The pathogenesis of GSD is still unclear. The more accepted theories are vascular and lymphatic proliferation and enhanced osteoclast activity [5]. Impact or trauma may be its inducing factors. The disease can occur at any age, but it is more common in children and adolescents. Its pathological characteristics include obvious proliferation and expansion of lymphatic vessels and progressive osteolysis [6]. GSD can affect bones throughout the body, and the extent of osteolysis varies greatly. Some patients invade only a single bone, while others suffer from large-scale osteolysis, and the affected bones can be completely absorbed [7, 8]. Little new bone formation occurs after the disease stabilizes, although there are case reports of patients with mild bone formation [9]. The clinical manifestations of patients are diverse, mainly depending on the affected parts, such as pain, swelling, dyspnea, chylothorax, pathological fractures, and limited activity. In severe cases, it may even lead to patient death. Patients with GSD often require comprehensive treatment and long-term follow-up, which seriously affects their quality of life.

Due to the rarity and complexity of GSD, its clinical diagnosis and treatment are very difficult. Fortunately, recent clinical and scientific discoveries have enhanced our knowledge of the genetics and pathophysiology of this disease, and targeted drugs such as sirolimus are being used to treat patients with GSD. Therefore, this study aimed to screen the literature on GSD from 2013 to 2023, and analyze its clinical characteristics and treatment methods by integrating the data of GSD patients. We also discuss the current management model of GSD patients based on our research results, hoping to provide experience and help to clinicians.

## Materials and methods

This study followed the PRISMA 2020 statement guidelines [10]. The PRISMA checklist of items included in this systematic review is available in Additional file 1: Table S1.

### Search strategy

A systematic search was conducted for all literature on GSD published in PubMed, Web of Science, Embase and the Cochrane Library from January 1, 2013 to January 1, 2023, and the clinical information of all GSD patients was collected. The keywords we used included “Gorham-Stout disease”, “Gorham Stout disease”, “Gorham disease”, “Gorham’s disease”, “Gorham-Stout syndrome”, “Gorham Stout syndrome”, “Gorham’s syndrome”, “Gorham syndrome “, “disappearing bone disease”, “vanishing bone disease”, “massive osteolysis”, “idiopathic massive osteolysis” and “essential osteolysis”. The subject words corresponding to each database were also added to the search terms. We also screened the reference lists of the included studies to identify additional literature on GSD.

### Inclusion and exclusion criteria

We included only articles written in English and the content of all the articles was checked to avoid duplication of case reports. The included literature needed to contain clinical information on the GSD patients. Although some articles did not provide all the patient-related information, we also included them in the study. Based on other authors’ articles and guidelines [11–15], we summarized the following four criteria to help include patients with GSD: [1] lesion tissue biopsy showing lymphatic dilation (positive PROX-1 and D2-40 staining) and no cellular atypia; [2] evidence of bone destruction on imaging; [3] local progressive bone (cortical and medullary) resorption with little or no osteogenic reaction; and [4] no positive evidence of genetic, metabolic, tumor, immune, or infection. Literature and data lacking any points that prevented patients from being clearly diagnosed with GSD were excluded. In addition, conference abstracts and comments or responses to articles were also excluded.

### Literature screening

**Fig. 1:**
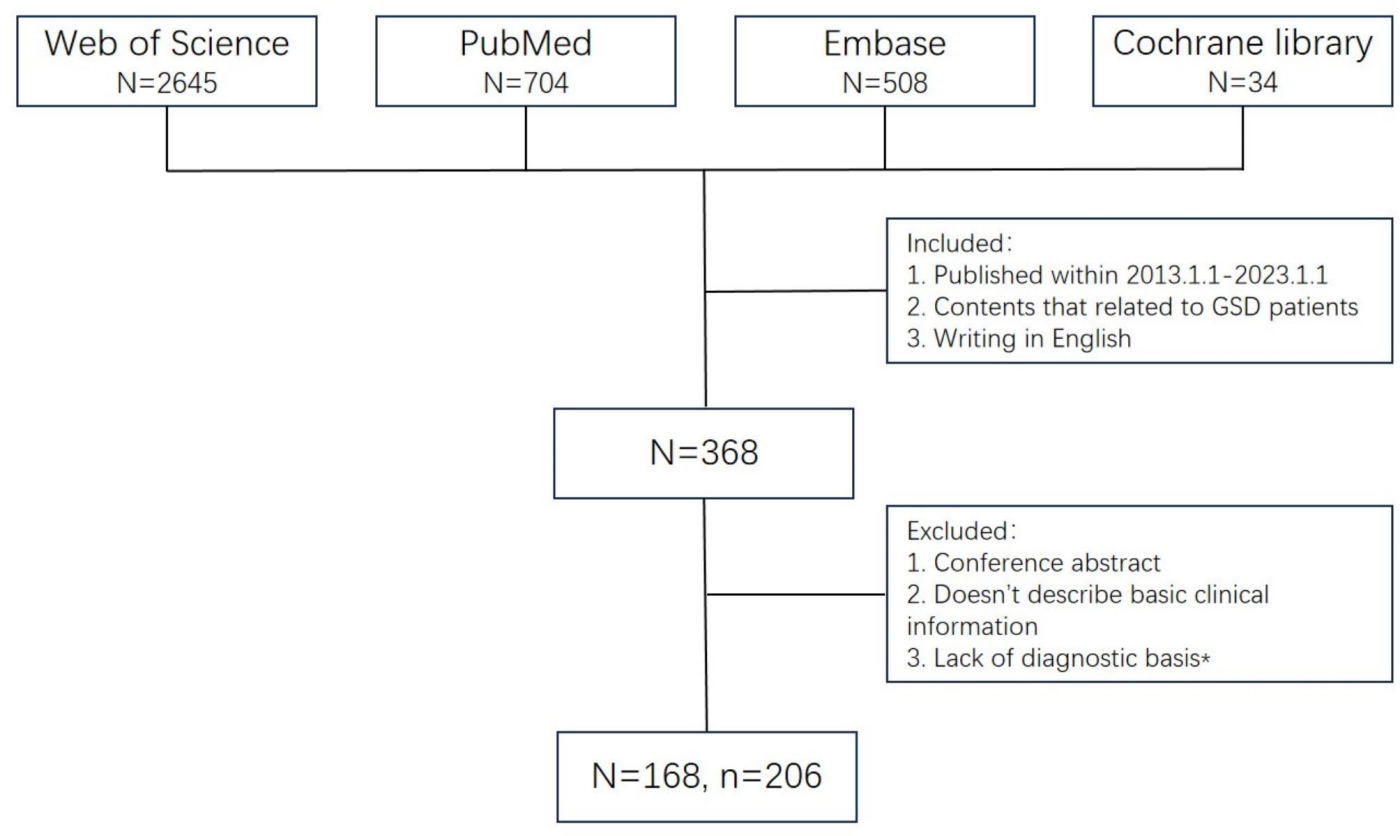
Screening process for search studies. N: the number of articles, n: the number of patients, *: the four criteria we described in the method

The literature screened from the four databases through the above search strategy was screened by two authors according to the prescribed inclusion and exclusion criteria (Fig. 1). The two authors independently read the full texts of the included studies and extracted patient clinical information. Any objections to the included literature or extracted clinical information will be discussed and resolved by a third author.

### Definition

The age of onset was defined as the age at which patients first presented with GSD-related symptoms (such as pain, fracture, swelling, etc.) or incidentally found GSD-related imaging findings (such as bone destruction, etc.). The age of diagnosis was defined as the age at which GSD was diagnosed by pathological biopsy. The sites of the disease were classified according to different bones and organs. Single osteolysis was defined as involvement of only one bone, and multiple osteolysis was defined as involvement of two or more bones. The appendiceal bones include the upper and lower limb bones, and the axial bones include the skull, ribs, vertebrae, and sternum.

### Data extraction

The following data were extracted: patient’s sex, age at onset, age at diagnosis, bone involvement, visceral involvement, clinical manifestations, treatment methods and prognosis. Data extraction was performed independently by two authors, and discrepancies were discussed and resolved by a third author. Missing values were marked as NR, and patient data with missing values were excluded in subsequent analyses.

### Quality assessment

Two authors assessed the quality of the included case reports/case series using the CARE guidelines [16]. Due to heterogeneity in the data among the included studies, no further meta-analysis was performed.

### Statistical analysis

Quantitative data were analyzed descriptively using mean, standard deviation (SD) and percentage values. Comparisons between two groups were performed using the chi-square test or Fisher’s exact probability test. Logistic regression was used to analyze factors affecting patient death. *P* < 0.05 indicated a statistically significant difference. All the data were analyzed statistically using SPSS 26.0.

## Results

**Fig. 2:**
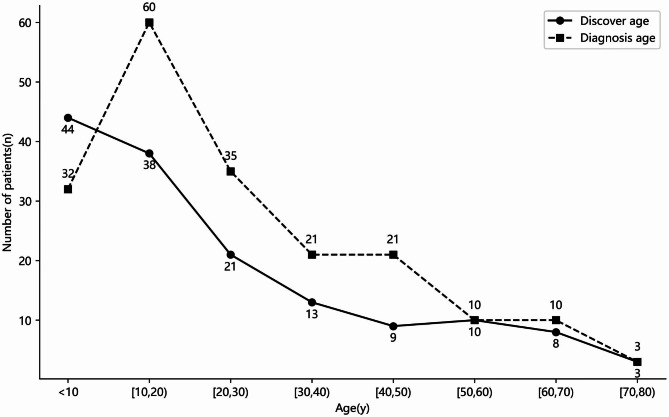
Distribution of patients by age at onset and diagnosis

**Fig. 3:**
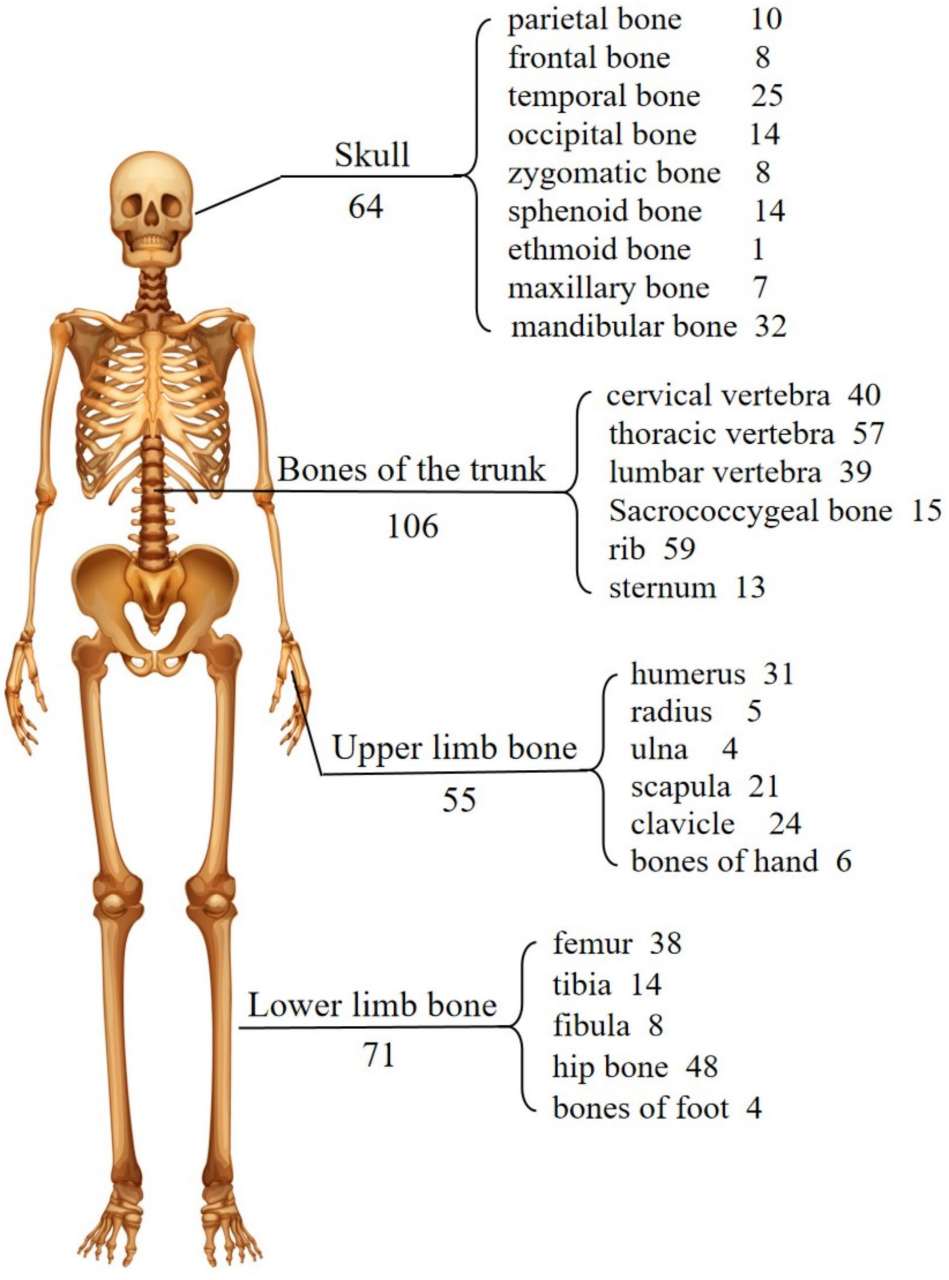
Distribution of bony involvement in GSD

Overall, 72.52% of case reports/case series met the quality criteria (Additional file 1: Table S2), and the included literature was generally of satisfactory quality in terms of the patients’ clinical data. Table 1 lists the demographic and clinical characteristics of the 206 GSD patients included in the study, including 119 males, 81 females and 6 patients with unknown sex. There appears to be a slight male predominance (with a ratio of 1.47:1) in GSD patients. The age of onset of patients was widely distributed, ranging from 0 to 77 years old (Figs. 2), 15 (10.3%) patients developed symptoms before 1 year of age, and 74 (50.7%) patients developed symptoms before 18 years old. Two patients were diagnosed with lymphatic malformation during the fetal period and were further diagnosed with GSD by biopsy after birth. In addition, the average time from onset to diagnosis was 3.5 years, and one patient even received a diagnosis of GSD 31 years after onset. Any bone in patients with GSD may suffer from symptoms of osteolysis. Figure 3 shows the number of patients with osteolysis in each bone of the body. The number of patients with osteolysis in the axial bone was greater than that in the appendiceal bone (154/206 vs. 106/206, 74.8% vs. 51.5%, *P* < 0.05), and the number of patients with multiple osteolytic lesions was greater than that with single osteolytic lesions (77.2% vs. 22.8%). Interestingly, the mandible accounted for the largest proportion of patients with single osteolytic lesions (19/47, 40.4%). Overall, GSD was more likely to occur in the spine (46.1%), ribs (28.6%), hip (23.3%), femur (18.4%), mandible (15.5%), and humerus (15.0%). In addition to affecting the bones of the body, GSD may also affect the lungs, liver, spleen, other internal organs and soft tissues of the body. Eleven (5.3%) patients had lung involvement, and 11 (5.3%) patients had spleen involvement. Interestingly, the splenic lesions were all asymptomatic.

**Table 1:** Demographic, clinical features and treatment of GSD patients

Variables	
Number	206
Sex, n (%)	
Male Female Unknown	119 (59.5%) 81 (40.5%) 6
Age (years), mean ± SD (range)	
Onset time Diagnosis time Duration	22.7 ± 19.8 (0-77, *n*=146) 25.6 ± 18.0 (0.17-77, *n*=192) 3.5 ± 5.2 (0-31, *n*=139)
Involvement, n (%)	
Monostotic Polyostotic	47 (22.8%) 159 (77.2%)
Symptoms, n (%)	
Pain Pathological fracture Swelling Dyspnea Chylothorax Nervous system impairment CSF leakage Hearing impairment	141 (68.4%) 53 (25.7%) 43 (20.9%) 40 (19.4%) 39 (18.9%) 38 (18.4%) 14 (6.8%) 6 (2.9%)
Treatment, n (%)	
Surgery Bisphosphonates Sirolimus Interferon	121 (66.9%) 103 (56.9%) 33 (18.2%) 32 (17.7%)
Quality of life, n (%)	
PR SD PD Dead Unknown	92 (44.7%) 17 (8.3%) 11 (5.3%) 14 (6.8%) 72 (35.0%)
Imaging evaluation, n (%)	
PR SD PD Dead Unknown History of previous trauma, n (%)	14 (6.8%) 74 (35.9%) 18 (8.7%) 14 (6.8%) 86 (41.7%) 35 (17.0%)

CSF: Cerebral Spinal Fluid; PR: Partial Response; SD; Stable Disease; PD: Progressive Disease

Pain is the most common symptom/complication of GSD patients, with 68.4% of patients complaining of pain in the lesion area. In addition, 25.7% and 20.9% of patients presented with pathological fractures and swelling in the lesion area respectively. Another common complication of GSD is pleural effusion. Among the GSD patients in the past ten years, a total of 56 patients reported pleural effusion, of whom 39 patients were clearly diagnosed with chylothorax. Moreover, 6 patients were found to have peritoneal effusion, 4 of whom were clearly chylous, and pericardial effusion was found in 5 patients. When the spine is involved, spinal cord compression can occur, and 38 (18.4%) patients experienced varying degrees of nervous system impairment. In addition, 14 (6.8%) patients presented with cerebrospinal fluid leakage caused by skull base osteolysis, and 9 (64.3%) of them experienced symptoms of meningitis.

There are various treatment methods mentioned in the literature, which are generally divided into four categories: surgery, drugs, radiotherapy and physical rehabilitation (Table 1). Surgery (66.9%) and bisphosphonates (56.9%) are still the mainstream treatment methods. A total of 33 (18.2%) patients had used sirolimus. The specific treatment measures are shown in Additional file 1: Table S3. Although there is no complete cure for GSD, the current treatment model is still meaningful. According to the assessment of quality of life, 92 (44.7%) patients experienced relief of clinical symptoms and improved quality of life after treatment. According to imaging evaluation, 74 (35.9%) patients had stable lesions after treatment. Furthermore, no patients have been reported to develop osteonecrosis of the jaw due to bisphosphonate use. Among the 14 patients who died, 5 died of respiratory failure caused by pleural effusion, 4 died of severe infection, 4 died of spinal cord compression, and 1 died of severe infection combined with respiratory failure. The mortality rate among patients with chylothorax was 12.8%. Through logistic regression analysis, we found that pleural effusion was a risk factor for patient death (*P* < 0.05) (Table 2).

**Table 2:** Univariate regression analysis of the risk factors for mortality

Variables	OR (95%CI)	*P*
**Age**	1.01 (0.98 - 1.04)	0.679
**Sex**		
Female	1 (ref)	
Male	1.30 (0.37 - 4.53)	0.678
**Chylothorax**		
Without	1 (ref)	
With	2.15 (0.67 - 6.88)	0.198
**Pleural effusion**		
Without	1 (ref)	
With	3.30 (1.08 - 10.09)	**0.036**
**Involvement**		
Monostotic	1 (ref)	
Polyostotic	38606352.74 (0.00 - inf)	0.993

## Discussion

However, due to the limited number of patients with GSD, high-quality research in this field is lacking. Scientifically describing the clinical characteristics of patients with GSD is important because it provides information that can improve diagnostic accuracy and help improve treatment options. This study is currently the largest database of biopsy-diagnosed GSD patients. We clarified the clinical manifestations, treatment status and prognosis of GSD patients through data analysis and provided experience for the diagnosis and treatment of patients.

Most patients require a long period of time from onset to diagnosis. This is due both to unfamiliarity with the clinical manifestations of GSD and the lack of diagnostic standards. Many patients often require discussion by a multidisciplinary team to obtain a final diagnosis [17]. The diagnosis of GSD may be very difficult and requires a combination of the patient’s clinical symptoms, imaging findings, and pathological results, and the possibility of other diseases also needs to be excluded [12, 13]. In the 1980s, Heffez et al. proposed eight criteria that can be used for the diagnosis of GSD [11]. Although the seventh criterion, “absence of visceral involvement”, now seems inappropriate [15], the other seven criteria can still be used to assist in the diagnosis of GSD.

GSD may have a greater incidence in males and may occur at any age, but the incidence is greater in childhood. Past experience has shown that its typical manifestations are pain, swelling and pathological fractures in the lesion area, which can lead to functional impairment. Our statistical data further confirmed this view. The pain can occur spontaneously or be caused by pathological fractures [14]. Chylothorax is a serious complication of GSD, and it has been reported that the mortality rate of patients with chylothorax is approximately 43.6% [18, 19], which is significantly greater than that of patients without chylothorax, while the mortality rate of patients with chylothorax in our study was 12.8%. This may be due to changes in treatment patterns over the past decade that have improved patient prognosis or reporting bias. Our study showed that pleural effusion, but not chylothorax, is a risk factor for patient death. This may be because some scholars did not further analyze the nature of pleural effusion or simply described chylothorax as pleural effusion, which biased the results. The mechanism of chylothorax is that irregular lymphatic vessels directly invade the pleura from the ribs or spine, and chylous fluid flows into the chest cavity from the thoracic duct or retroperitoneum. Dynamic contrast-enhanced magnetic resonance lymphangiography can be very effective for revealing the presence and nature of abnormal pulmonary lymphatic flow [19–21].

The characteristic imaging manifestation of GSD is progressive osteolysis accompanied by cortical bone destruction. As our results show, this sign may be found in any bone. Some scholars have summarized the imaging manifestations of bone involvement in GSD into four stages: the first stage, in which “patchy” signal shadow changes can be observed in the bone marrow; the second stage, in which “patchy” signal shadows converge in the bone marrow, and focal or diffuse signal changes in the bone marrow can be observed; the third stage, in which bone cortical destruction with adjacent soft tissue invasion occurs; and the fourth stage, in which osteolysis disappears, and the lesions are replaced by fibrous tissue or surrounding soft tissue [22]. Expert consensus recommends that patients with suspected GSD first undergo MRI for screening [12], and MRI can be used to evaluate the degree of involvement of bones, soft tissues and organs. The lesion and surrounding infiltrative soft tissue show low signal intensity on T1-weighted images and high signal intensity on T2-weighted images [23]. CT is mainly used to evaluate the degree of bone destruction in patients. Loss of medullary substance and bone cortex can be observed at the lesion site. At the same time, three-dimensional CT reconstruction helps doctors judge whether the patient can undergo surgery and the method of surgery, and it can also be used to display changes in the patient’s osteolytic area during follow-up [24–26]. Expert consensus states that pathological biopsy is necessary for the diagnosis of GSD [12], which is one of the reasons why we used a positive pathological biopsy as an inclusion criterion. Typical histological images show abnormal expansion of lymphatic vessels and thin-walled blood vessels, loss of cortex and trabecular bone, and positive staining for PROX-1 and D2-40 [13], and increased activity of osteoclasts can sometimes be observed [27]. Laboratory tests of GSD patients are often unremarkable, although some patients have been reported to have elevated alkaline phosphatase levels [28, 29]. However, laboratory test results are also important, and we can use these results to rule out the possibility of many diseases.

**Fig. 4:**
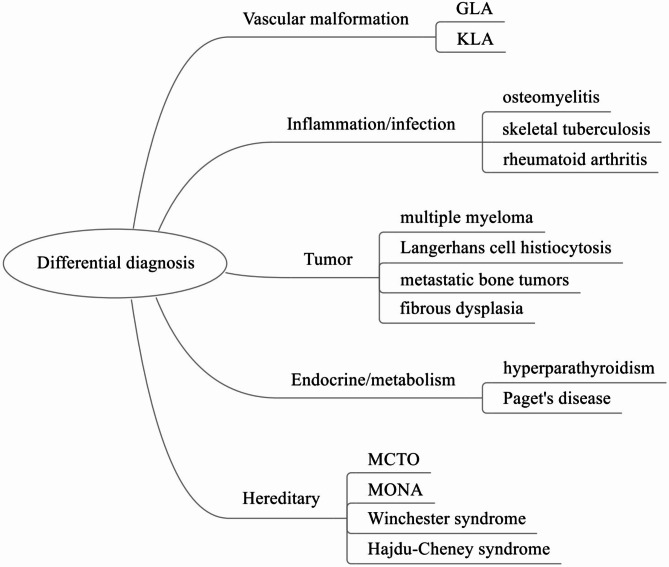
Differential diagnosis of GSD. GLA: Generalized lymphatic anomaly, KLA: Kaposiform lymphangiomatosis, MCTO: Multicentric carpotarsal osteolysis syndrome, MONA: Multicentric Osteolysis, Nodulosis, and Arthropathy

Although GSD is a rare disease, its typical clinical symptoms are not specific. Doctors who do not specialize in this field can easily confuse GSD with other diseases. The differential diagnosis of GSD includes vascular malformations, inflammation/infection, tumors, metabolism, genetics and other diseases (Fig. 4). By reading the literature published in the past, we analyzed and summarized some frequently mentioned diseases (Table 3). All in all, we believe that GSD should be considered when a patient presents with symptoms of osteolysis.

**Table 3:** Differential diagnosis of GSD

Diseases	Distinguishing features from GSD
GLA	GLA is characterized by multifocal lymphatic malformations involving bones, viscera, and soft tissues. The osteolytic lesions are confined to the medullary cavity and are relatively stable.
KLA	KLA can cause thrombocytopenia syndrome and coagulation dysfunction, and spindle-shaped “Kaposi-like” endothelial cells can be observed via histological biopsy.
Osteomyelitis	Patients have elevated ESR and CRP. Biopsy and bacterial culture of lesion tissue can help identify the infecting microorganisms.
Skeletal tuberculosis	Patients may have symptoms such as low fever and night sweats, and may also have pulmonary tuberculosis. They generally have a history of tuberculosis infection/contact, and tuberculin tests and tuberculosis cultures are usually positive.
Rheumatoid arthritis	Patients will experience morning stiffness. RF, ACPA, CRP or ESR are generally elevated, and X-rays can reveal characteristic joint changes.
Multiple myeloma	Typical symptoms of multiple myeloma are hypercalcemia, renal failure, anemia, and bone pain. M-protein is elevated in patients, and bone marrow biopsy may reveal clonal plasma cells or plasma cell tumors.
Langerhans cell histiocytosis	Langerhans cell histiocytosis may present with fever, rash, lymphadenopathy, etc. Pathological biopsy shows well-differentiated histiocytic proliferation, with positive CD68, CD1a, S100, and CD207. Birbeck granules can be observed.
Metastatic bone tumors	Patients have primary tumor lesions. PET-CT, tumor markers and pathological biopsy can assist in diagnosis.
Fibrous dysplasia	X-rays show fibrous dysplasia as ground-glass, and blood tests may show elevated ALP. Some patients have GNAS mutations.
Hyperparathyroidism	The patient’s serum calcium and PTH are elevated, and parathyroid adenoma can be detected in most patients. In rare cases, a complication called brown tumor may occur.
Paget’s disease	Blood tests may show elevated ALP. Some patients have SQSTM1 and RANK mutations.
MCTO	It is characterised by carpal-tarsal destruction and kidney failure. The disease results from mutations in the MAFB gene.
MONA	The bone abnormalities begin in the hands and feet and then spread to other parts of the body, with many patients developing subcutaneous nodules. The disease results from mutations in the MMP2 gene.
Winchester syndrome	The clinical presentation is similar to MONA, but without subcutaneous nodules. The disease results from mutations in the MMP14 gene.
Hajdu-Cheney syndrome	Hajdu-Cheney syndrome causes problems with the patient’s connective tissue, which can result in growth retardation, hearing loss, osteopenia, and hirsutism. The disease results from mutations in the NOTCH2 gene.

GLA: Generalized lymphatic anomaly, KLA: Kaposiform lymphangiomatosis, ESR: Erythrocyte sedimentation rate, CRP: C-reactive protein, RF: Rheumatoid factor, ACPA: Anti-cyclic peptide containing citrulline, ALP: Alkaline Phosphatase, MCTO: Multicentric carpotarsal osteolysis syndrome, MONA: Multicentric Osteolysis, Nodulosis, and Arthropathy

The treatment of GSD mainly focuses on symptomatic treatment and delaying the progression of bone disease, with the purpose of reducing complications and improving patient quality of life. Since GSD was first reported, many treatments including surgery, drugs, and radiation therapy have been attempted. Surgical treatment mainly includes lesion resection, prosthesis or bone grafting and reconstruction, and thoracic puncture drainage. The indications for surgery are still unknown, and are mostly determined based on the patient’s condition and the experience of the attending physician. Past experience suggests that surgical treatment should be performed during the stable phase of osteolysis to prevent further osteolysis after surgery [30]. In contrast to those of ordinary orthopedic diseases, the progressive osteolytic characteristics of GSD may lead to surgical failure, such as further dissolution of the grafted bone, graft nonunion, implant fracture and other postoperative complications. Therefore, many patients require multiple additional surgeries to achieve the desired results [31–34]. Radiation therapy can inhibit angiogenesis and reduce the size of lesions and is often used in combination with surgery or drugs. Guidelines recommend the use of conventional fractionated radiotherapy for patients with symptomatic GSD, with a total dose ranging from 36 to 45 Gy [35]. Traditional drug treatments mainly include bisphosphonates, interferon, calcium and vitamin D. Bisphosphonates inhibit osteolysis by inhibiting osteoclast activity and are often used in combination with calcium and vitamin D. IFN-α2b (interferon alpha-2b) is an immunosuppressant that aims to reduce angiogenesis-induced osteolysis through its antiangiogenic effect [36, 37].

Currently, the mortality rate of GSD patients is unclear. Ozeki et al. investigated the prognosis of 41 GSD patients through questionnaires and reported that the mortality rate of GSD patients was less than 10% [38]. Angelini et al. published a review that summarized GSD patients from 1955 to 2021 retrieved from PubMed [14]. Unfortunately, we found that the mortality rate of GSD patients from 2003 to 2012 was not significantly different from that of GSD patients in our study. Due to the limitations of the study we were unable to compare whether changes in treatment modalities and the use of targeted drugs had any effect on GSD patients, and more high-quality research is needed to provide additional evidence.

With continuous research on the pathogenesis of vascular diseases, sirolimus (an mTOR inhibitor) has gradually been discovered for its ability to treat various vascular diseases. Past research has shown that the PI3K-Akt-mTOR signaling pathway is important for regulating the formation of blood vessels and lymphatic vessels. Moreover, mTOR inhibitors can block downstream protein synthesis and have antitumor and antiangiogenic effects [39]. Kaposiform hemangioendothelioma is a complex and severe vascular disease similar to GSD, and previous studies by our team have shown that sirolimus has a significant effect on the treatment of this disease [40, 41]. The first case of sirolimus in the treatment of GSD that we were able to retrieve was reported in 2011 [42], and an increasing number of clinical studies have proven the effectiveness of sirolimus, which can not only shrink tumors but also improve patients’ quality of life [43–45]. In addition, research results indicate that somatic activating mutations in KRAS may be associated with GSD and suggest that trametinib (a MEK inhibitor) may be an effective drug for the treatment of GSD [46].

Researchers used to think that there were no lymphatic vessels in the bone, and it was speculated that the pathogenesis of GSD may be osteolysis caused by the proliferation of lymphatic vessels and blood vessels in the bone. Recent studies have revealed evidence of lymphatic vessels in human bones, and the formation of lymphatic vessels can stimulate hematopoiesis and bone regeneration [47]. This discovery may provide more insight into the mechanism and treatment of GSD.

### Limitation

Most of the literature included in this study were case reports or case series reports, lacking high-quality research. The conclusions of this study were based on case data extracted from existing literature, and the number of doctors who can recognize and diagnose GSD is very limited, so there is reporting bias.

## Conclusion

GSD is an extremely rare disease with an unknown etiology. The natural course of this disease is still unclear, and the treatment methods vary clinically. GSD not only has a high mortality rate, but also seriously affects the quality of life of patients. We reviewed the literature from the past decade to summarize the common clinical manifestations, treatment methods and prognoses of GSD patients. At the same time, we discussed the diagnostic strategy for GSD and the diseases that need to be identified based on the literature. Despite the emergence of new treatments such as sirolimus, the treatment of GSD still needs further research.

## Electronic supplementary material

Below is the link to the electronic supplementary material.

Supplementary Material 1

## Data Availability

The datasets generated and/or analyzed during the current study are available from the corresponding author on reasonable request.

## Abbreviations

GSD: Gorham-Stout disease
GLA: Generalized lymphatic anomaly
KLA: Kaposiform lymphangiomatosis
