# Supplementary material for: Clinical features and current management experience in Gorham-Stout disease: a systematic review
Source: Orphanet J Rare Dis. 2025 Mar 19;20:134. doi: 10.1186/s13023-025-03649-9 (PMC11921740; doi:10.1186/s13023-025-03649-9)
Supplement: Supplementary file 1 — Supplementary Material 1 [file 13023_2025_3649_MOESM1_ESM.docx]

**Supplementary Material**

**Table 1. PRISMA checklist of items included in this systematic review.**

| **Section and Topic** | **Item #** | **Checklist item** | **Location where item is reported** |
| --- | --- | --- | --- |
| **TITLE** | | |  |
| Title | 1 | Identify the report as a systematic review. | 1 |
| **ABSTRACT** | | |  |
| Abstract | 2 | See the PRISMA 2020 for Abstracts checklist. | 2,3 |
| **INTRODUCTION** | | |  |
| Rationale | 3 | Describe the rationale for the review in the context of existing knowledge. | 4 |
| Objectives | 4 | Provide an explicit statement of the objective(s) or question(s) the review addresses. | 5 |
| **METHODS** | | |  |
| Eligibility criteria | 5 | Specify the inclusion and exclusion criteria for the review and how studies were grouped for the syntheses. | 5,6 |
| Information sources | 6 | Specify all databases, registers, websites, organisations, reference lists and other sources searched or consulted to identify studies. Specify the date when each source was last searched or consulted. | 5 |
| Search strategy | 7 | Present the full search strategies for all databases, registers and websites, including any filters and limits used. | 5 |
| Selection process | 8 | Specify the methods used to decide whether a study met the inclusion criteria of the review, including how many reviewers screened each record and each report retrieved, whether they worked independently, and if applicable, details of automation tools used in the process. | 6 |
| Data collection process | 9 | Specify the methods used to collect data from reports, including how many reviewers collected data from each report, whether they worked independently, any processes for obtaining or confirming data from study investigators, and if applicable, details of automation tools used in the process. | 6,7 |
| Data items | 10a | List and define all outcomes for which data were sought. Specify whether all results that were compatible with each outcome domain in each study were sought (e.g. for all measures, time points, analyses), and if not, the methods used to decide which results to collect. | 7 |
|  | 10b | List and define all other variables for which data were sought (e.g. participant and intervention characteristics, funding sources). Describe any assumptions made about any missing or unclear information. | 7 |
| Study risk of bias assessment | 11 | Specify the methods used to assess risk of bias in the included studies, including details of the tool(s) used, how many reviewers assessed each study and whether they worked independently, and if applicable, details of automation tools used in the process. | NA |
| Effect measures | 12 | Specify for each outcome the effect measure(s) (e.g. risk ratio, mean difference) used in the synthesis or presentation of results. | NA |
| Synthesis methods | 13a | Describe the processes used to decide which studies were eligible for each synthesis (e.g. tabulating the study intervention characteristics and comparing against the planned groups for each synthesis (item #5)). | NA |
|  | 13b | Describe any methods required to prepare the data for presentation or synthesis, such as handling of missing summary statistics, or data conversions. | NA |
|  | 13c | Describe any methods used to tabulate or visually display results of individual studies and syntheses. | NA |
|  | 13d | Describe any methods used to synthesize results and provide a rationale for the choice(s). If meta-analysis was performed, describe the model(s), method(s) to identify the presence and extent of statistical heterogeneity, and software package(s) used. | NA |
|  | 13e | Describe any methods used to explore possible causes of heterogeneity among study results (e.g. subgroup analysis, meta-regression). | NA |
|  | 13f | Describe any sensitivity analyses conducted to assess robustness of the synthesized results. | NA |
| Reporting bias assessment | 14 | Describe any methods used to assess risk of bias due to missing results in a synthesis (arising from reporting biases). | NA |
| Certainty assessment | 15 | Describe any methods used to assess certainty (or confidence) in the body of evidence for an outcome. | NA |
| **RESULTS** | | |  |
| Study selection | 16a | Describe the results of the search and selection process, from the number of records identified in the search to the number of studies included in the review, ideally using a flow diagram. | 6 |
|  | 16b | Cite studies that might appear to meet the inclusion criteria, but which were excluded, and explain why they were excluded. | NA |
| Study characteristics | 17 | Cite each included study and present its characteristics. | 9 |
| Risk of bias in studies | 18 | Present assessments of risk of bias for each included study. | NA |
| Results of individual studies | 19 | For all outcomes, present, for each study: (a) summary statistics for each group (where appropriate) and (b) an effect estimate and its precision (e.g. confidence/credible interval), ideally using structured tables or plots. | 7 |
| Results of syntheses | 20a | For each synthesis, briefly summarise the characteristics and risk of bias among contributing studies. | NA |
|  | 20b | Present results of all statistical syntheses conducted. If meta-analysis was done, present for each the summary estimate and its precision (e.g. confidence/credible interval) and measures of statistical heterogeneity. If comparing groups, describe the direction of the effect. | NA |
|  | 20c | Present results of all investigations of possible causes of heterogeneity among study results. | NA |
|  | 20d | Present results of all sensitivity analyses conducted to assess the robustness of the synthesized results. | NA |
| Reporting biases | 21 | Present assessments of risk of bias due to missing results (arising from reporting biases) for each synthesis assessed. | NA |
| Certainty of evidence | 22 | Present assessments of certainty (or confidence) in the body of evidence for each outcome assessed. | NA |
| **DISCUSSION** | | |  |
| Discussion | 23a | Provide a general interpretation of the results in the context of other evidence. | 9 |
|  | 23b | Discuss any limitations of the evidence included in the review. | 14 |
|  | 23c | Discuss any limitations of the review processes used. | 14 |
|  | 23d | Discuss implications of the results for practice, policy, and future research. | 14 |
| **OTHER INFORMATION** | | |  |
| Registration and protocol | 24a | Provide registration information for the review, including register name and registration number, or state that the review was not registered. | NA |
|  | 24b | Indicate where the review protocol can be accessed, or state that a protocol was not prepared. | NA |
|  | 24c | Describe and explain any amendments to information provided at registration or in the protocol. | NA |
| Support | 25 | Describe sources of financial or non-financial support for the review, and the role of the funders or sponsors in the review. | 16 |
| Competing interests | 26 | Declare any competing interests of review authors. | 17 |
| Availability of data, code and other materials | 27 | Report which of the following are publicly available and where they can be found: template data collection forms; data extracted from included studies; data used for all analyses; analytic code; any other materials used in the review. | 8 |

**Table 2. Quality assessment of the case reports/case series included in this systematic review.**

| Authors and year | Yes | No | N/A | % yes |
| --- | --- | --- | --- | --- |
| Asha, M. L., et al. (2013). Gerodontology 30(4): 296-302. | 19 | 5 | 6 | 63.33 |
| Barman, A., et al. (2013). NeuroRehabilitation 33(1): 121-126. | 25 | 5 | 0 | 83.33 |
| Chen, B., et al. (2013). Experimental and Therapeutic Medicine 5(4): 1017-1018. | 23 | 5 | 2 | 76.67 |
| Dong, Q., et al. (2013). Experimental and Therapeutic Medicine 5(1): 162-164. | 20 | 4 | 6 | 66.67 |
| Esmailiejah, A. A., et al. (2013). Archives of Iranian Medicine 16(11): 686-690. | 17 | 10 | 3 | 56.67 |
| Huang, S. Y., et al. (2013). Respiratory Care 58(11): e144-e148. | 20 | 10 | 0 | 66.67 |
| Kilicoglu, Z. G., et al. (2013). Spine Journal 13(5): e11-e14. | 20 | 9 | 1 | 66.67 |
| Leite, I., et al. (2013). Pediatric Dermatology 30(3): 374-378. | 21 | 9 | 0 | 70 |
| Liu, C. Y., et al. (2013). Thoracic Cancer 4(2): 207-211. | 25 | 5 | 0 | 83.33 |
| Maruani, A., et al. (2013). Pediatric Dermatology 30(3): 391-394. | 17 | 12 | 1 | 56.67 |
| Morimoto, N., et al. (2013). International Journal of Pediatric Otorhinolaryngology 77(9): 1596-1600. | 22 | 7 | 1 | 73.33 |
| Noda, M., et al. (2013). General Thoracic and Cardiovascular Surgery 61(6): 356-358. | 21 | 8 | 1 | 70 |
| Ozbayrak, M., et al. (2013). Korean Journal of Radiology 14(6): 946-950. | 23 | 6 | 1 | 76.67 |
| Ravindran, R. and A. Karunakaran (2013). Case Rep Dent 2013: 723583. | 21 | 8 | 1 | 70 |
| Sekharappa, V., et al. (2013). Asian Spine J 7(3): 242-247. | 21 | 9 | 0 | 70 |
| Situma, M., et al. (2013). Journal of Pediatric Surgery 48(1): 239-242. | 25 | 5 | 0 | 83.33 |
| Stojsic, J., et al. (2013). Journal of Orthopaedic Science 18(1): 186-189. | 14 | 10 | 6 | 46.67 |
| Tavakoli Darestani, R., et al. (2013). Trauma Mon 18(1): 41-45. | 20 | 9 | 1 | 66.67 |
| Tripathy, S. K., et al. (2013). Orthopaedic Surgery 5(1): 72-76. | 21 | 8 | 1 | 70 |
| Choi, B. S., et al. (2014). Pediatr Gastroenterol Hepatol Nutr 17(1): 52-56. | 23 | 6 | 1 | 76.67 |
| Coulter, I. C., et al. (2014). Clinical Neurology and Neurosurgery 116: 83-86. | 18 | 7 | 5 | 60 |
| Gem, M., et al. (2014). Acta Orthopaedica Et Traumatologica Turcica 48(4): 467-471. | 21 | 9 | 0 | 70 |
| Kakhaki, A. D., et al. (2014). Iranian Red Crescent Medical Journal 16(11). | 24 | 6 | 0 | 80 |
| Posadas, M. D., et al. (2014). European Review for Medical and Pharmacological Sciences 18(1): 81-83. | 17 | 7 | 6 | 56.67 |
| Scheller, K., et al. (2014). Oral and maxillofacial surgery 18(1): 75-79. | 23 | 5 | 2 | 76.67 |
| Suero Molina, E. J., et al. (2014). J Neurosurg Spine 21(6): 956-960. | 24 | 6 | 0 | 80 |
| Alves, V. M., et al. (2015). Nucl Med Rev Cent East Eur 18(2): 97-101. | 23 | 6 | 1 | 76.67 |
| Baud, J., et al. (2015). BMC research notes 8: 333. | 24 | 6 | 0 | 80 |
| Carbó, E., et al. (2015). European Spine Journal. | 23 | 6 | 1 | 76.67 |
| Davalos, E. A., et al. (2015). Radiology Case Reports 10(2). | 13 | 11 | 6 | 43.33 |
| Duraisamy, D., et al. (2015). J Clin Diagn Res 9(11): Zd25-27. | 21 | 7 | 2 | 70 |
| Findler, M., et al. (2015). Lymphatic Research and Biology 13(1): 62-65. | 21 | 7 | 2 | 70 |
| Gulati, U., et al. (2015). J Maxillofac Oral Surg 14(3): 548-557. | 23 | 6 | 1 | 76.67 |
| Karim, S. M., et al. (2015). American journal of orthopedics (Belle Mead, N.J.) 44(11): E473-E477. | 23 | 7 | 0 | 76.67 |
| Kim, B. J., et al. (2015). Korean J Thorac Cardiovasc Surg 48(1): 90-94. | 22 | 5 | 3 | 73.33 |
| Kim, M. K., et al. (2015). Journal of Oral and Maxillofacial Surgery 73(12): 2352-2360. | 22 | 6 | 2 | 73.33 |
| Kohno, M., et al. (2015). NMC Case Rep J 2(2): 80-84. | 22 | 7 | 1 | 73.33 |
| Kunjur, J., et al. (2015). Journal of Craniofacial Surgery 26(4): E302-E305. | 20 | 9 | 1 | 66.67 |
| Ohla, V., et al. (2015). BMC surgery 15: 24. | 24 | 5 | 1 | 80 |
| Pn, G., et al. (2015). J Orthop Case Rep 5(4): 30-33. | 23 | 6 | 1 | 76.67 |
| Rössler, J., et al. (2015). Journal of Pediatric Hematology/Oncology 37(8): e481-e485. | 25 | 4 | 1 | 83.33 |
| Sá, P., et al. (2015). Rev Bras Ortop 50(2): 239-242. | 18 | 9 | 3 | 60 |
| Shi, J., et al. (2015). Skeletal Radiology 44(7): 1033-1038. | 20 | 9 | 1 | 66.67 |
| Yerganyan, V. V., et al. (2015). Journal of Bone Oncology 4(2): 42-46. | 21 | 9 | 0 | 70 |
| Zhao, S. F., et al. (2015). The Journal of craniofacial surgery 26(2): e160-e162. | 24 | 5 | 1 | 80 |
| Amirjamshidi, A., et al. (2016). British Journal of Neurosurgery 30(6): 687-688. | 20 | 9 | 1 | 66.67 |
| Chan, C. K., et al. (2016). Malays Orthop J 10(3): 42-45. | 21 | 8 | 1 | 70 |
| Cramer, S. L., et al. (2016). Journal of Pediatric Hematology/Oncology 38(3): e129-e132. | 22 | 7 | 1 | 73.33 |
| Ellati, R., et al. (2016). European Review for Medical and Pharmacological Sciences 20(3): 426-432. | 22 | 8 | 0 | 73.33 |
| Evrenos, M. K., et al. (2016). J Maxillofac Oral Surg 15(Suppl 2): 335-338. | 20 | 9 | 1 | 66.67 |
| Ganal-Antonio, A. K., et al. (2016). Spine J 16(2): e67-75. | 24 | 5 | 1 | 80 |
| García, V., et al. (2016). Colombia Medica 47(4): 213-216. | 25 | 5 | 0 | 83.33 |
| Gataa, I. S., et al. (2016). Journal of Oral and Maxillofacial Surgery 74(9): 1774-1782. | 23 | 7 | 0 | 76.67 |
| Mukulchotrani, et al. (2016). Pravara Medical Review 8(3): 26-28. | 13 | 8 | 9 | 43.33 |
| Nozawa, A., et al. (2016). Pediatric Blood and Cancer 63(5): 931-934. | 22 | 8 | 0 | 73.33 |
| Yamagishi, E., et al. (2016). Journal of Pediatric Orthopaedics Part B 25(1): 48-53. | 23 | 6 | 1 | 76.67 |
| Bargagli, E., et al. (2017). AJP Reports 7(4): E226-E229. | 18 | 11 | 1 | 60 |
| Bocchialini, G., et al. (2017). International Journal of Surgery Case Reports 34: 110-114. | 23 | 6 | 1 | 76.67 |
| Jose Franco-Barrera, M., et al. (2017). Clinical Reviews in Allergy & Immunology 52(1): 125-132. | 23 | 7 | 0 | 76.67 |
| Kim, J. W., et al. (2017). Indian J Orthop 51(2): 217-221. | 20 | 9 | 1 | 66.67 |
| Liu, M., et al. (2017). Medicine (Baltimore) 96(42): e8184. | 22 | 4 | 4 | 73.33 |
| Mohapatra, M., et al. (2017). The Journal of clinical pediatric dentistry 41(6): 472-477. | 20 | 9 | 1 | 66.67 |
| Naqvi, A. A., et al. (2017). J Surg Case Rep 2017(2): rjx025. | 23 | 7 | 0 | 76.67 |
| Renacci, R. M. and R. J. Bartolotta (2017). Clinical Imaging 41: 83-85. | 21 | 8 | 1 | 70 |
| Sinha, R., et al. (2017). Iran J Pathol 12(3): 301-306. | 15 | 8 | 7 | 50 |
| Srivastava, S. K., et al. (2017). Indian J Orthop 51(1): 107-114. | 23 | 6 | 1 | 76.67 |
| Tateda, S., et al. (2017). Tohoku Journal of Experimental Medicine 241(4): 249-254. | 22 | 7 | 1 | 73.33 |
| Bruyère, C., et al. (2018). Journal of Solid Tumors 8(2): 20-24. | 18 | 6 | 6 | 60 |
| Costa, F. A. O., et al. (2018). Journal of Clinical and Diagnostic Research 12(5): PD17-PD19. | 23 | 6 | 1 | 76.67 |
| Ganau, M., et al. (2018). Hellenic Journal of Nuclear Medicine 21(3): 198-201. | 21 | 8 | 1 | 70 |
| Illeez, O. G., et al. (2018). Orthopade 47(12): 1032-1035. | 22 | 7 | 1 | 73.33 |
| Jaccard, A., et al. (2018). Surgical Neurology International 9(1). | 18 | 10 | 2 | 60 |
| Jatuworapruk, K., et al. (2018). International Journal of Rheumatic Diseases 21(7): 1458-1462. | 22 | 7 | 1 | 73.33 |
| Kotaru, A. C. and A. K. Rajput (2018). Journal of Bronchology and Interventional Pulmonology 25(4): 340-342. | 17 | 9 | 4 | 56.67 |
| Li, M.-h., et al. (2018). Orthopaedic Surgery 10(3): 276-280. | 24 | 6 | 0 | 80 |
| Liu, S., et al. (2018). Medicine (United States) 97(29). | 24 | 6 | 0 | 80 |
| Lova, F., et al. (2018). Radiology Case Reports 13(1): 96-100. | 18 | 6 | 6 | 60 |
| Maillot, C., et al. (2018). European spine journal 27(9): 2285-2290. | 24 | 5 | 1 | 80 |
| Mo, A. Z., et al. (2018). JBJS Case Connect 8(3): e70. | 26 | 4 | 0 | 86.67 |
| Noman, S., et al. (2018). Journal of the College of Physicians and Surgeons--Pakistan : JCPSP 28(9): S164-S165. | 14 | 8 | 8 | 46.67 |
| Reipschläger, M., et al. (2018). JAAD Case Reports 4(5): 458-461. | 19 | 10 | 1 | 63.33 |
| Soh, H. Y., et al. (2018). Oral Radiology 34(2): 179-184. | 19 | 4 | 7 | 63.33 |
| Stroh, I. G., et al. (2018). Journal of Neuro-Ophthalmology 38(1): 70-74. | 19 | 11 | 0 | 63.33 |
| Tasis, N., et al. (2018). Case Rep Pulmonol 2018: 2406496. | 20 | 10 | 0 | 66.67 |
| Yoshimoto, S., et al. (2018). Journal of Neurosurgery: Pediatrics 22(5): 508-512. | 24 | 6 | 0 | 80 |
| Ali, M., et al. (2019). Cureus 11(6): e4832. | 21 | 2 | 7 | 70 |
| Barbagli, G., et al. (2019). Interdisciplinary Neurosurgery: Advanced Techniques and Case Management 17: 79-83. | 24 | 6 | 0 | 80 |
| Du, C.-Z., et al. (2019). Quantitative Imaging in Medicine and Surgery 9(4): 565-578. | 18 | 9 | 3 | 60 |
| Feng, L., et al. (2019). BMC Musculoskeletal Disorders 20(1). | 21 | 6 | 3 | 70 |
| Jiang, Y., et al. (2019). Medicine (United States) 98(14). | 21 | 9 | 0 | 70 |
| Kaissi, A. A., et al. (2019). Medicines (Basel) 6(2). | 22 | 7 | 1 | 73.33 |
| Kim, J. H., et al. (2019). World Neurosurgery 129: 277-281. | 26 | 4 | 0 | 86.67 |
| Koto, K., et al. (2019). Molecular and Clinical Oncology 11(6): 551-556. | 25 | 4 | 1 | 83.33 |
| Matsumoto, T., et al. (2019). JBJS Case Connector 9(2). | 21 | 9 | 0 | 70 |
| Nozawa, A., et al. (2019). Internal Medicine 58(13): 1929-1933. | 24 | 6 | 0 | 80 |
| Simon, F., et al. (2019). Int J Pediatr Otorhinolaryngol 124: 152-156. | 22 | 7 | 1 | 73.33 |
| Tena-Sanabria, M. E., et al. (2019). BMC Pediatrics 19(1). | 27 | 3 | 0 | 90 |
| Wang, P., et al. (2019). BMC Musculoskeletal Disorders 20(1). | 27 | 3 | 0 | 90 |
| Zheng, C., et al. (2019). BMC Musculoskeletal Disorders 21(1). | 26 | 4 | 0 | 86.67 |
| Chang, K.-J., et al. (2020). Experimental and Therapeutic Medicine 19(6): 3851-3855. | 25 | 5 | 0 | 83.33 |
| Hosoya, M., et al. (2020). Journal of Otolaryngology - Head and Neck Surgery 49(1). | 26 | 4 | 0 | 86.67 |
| Hou, G., et al. (2020). Medicine (United States) 99(39): E22414. | 16 | 6 | 8 | 53.33 |
| Jagtap, R., et al. (2020). Oral Radiol 36(4): 389-394. | 27 | 2 | 1 | 90 |
| Jha, V. and A. Jha (2020). BMJ Case Reports 13(2). | 25 | 4 | 1 | 83.33 |
| Liang, Y., et al. (2020). BMC Musculoskeletal Disorders 21(1). | 27 | 2 | 1 | 90 |
| Rahman, N. A., et al. (2020). Journal of Taibah University Medical Sciences 15(2): 160-165. | 23 | 5 | 2 | 76.67 |
| Schneider, K. N., et al. (2020). Acta Orthopaedica 91(2): 209-214. | 22 | 8 | 0 | 73.33 |
| Stephens, S., et al. (2020). Journal of Clinical Neuroscience 72: 486-492. | 23 | 6 | 1 | 76.67 |
| Vaishya, R., et al. (2020). Journal of Orthopaedics 18: 177-180. | 19 | 9 | 2 | 63.33 |
| Yokoi, H., et al. (2020). Surgical Neurology International 11. | 21 | 8 | 1 | 70 |
| Duczkowski, M., et al. (2021). Polish journal of pathology : official journal of the Polish Society of Pathologists 72(2): 174-179. | 18 | 4 | 8 | 60 |
| Esper, A., et al. (2021). Oxford Medical Case Reports 2021(1): 8-12. | 20 | 8 | 2 | 66.67 |
| Evsyukov, A., et al. (2021). British Journal of Neurosurgery. | 25 | 4 | 1 | 83.33 |
| Grönroos, M. and A. Palomäki (2021). Journal of Medical Case Reports 15(1). | 24 | 5 | 1 | 80 |
| Kamble, P., et al. (2021). J Orthop Case Rep 11(1): 101-103. | 23 | 7 | 0 | 76.67 |
| Momanu, A., et al. (2021). Medicina (Kaunas, Lithuania) 57(7). | 24 | 5 | 1 | 80 |
| Rana, I., et al. (2021). Lymphology 54(4): 182-194. | 25 | 5 | 0 | 83.33 |
| Saify, F. Y., et al. (2021). J Oral Maxillofac Pathol 25(Suppl 1): S7-s10. | 18 | 5 | 7 | 60 |
| Saldarriaga, S. F., et al. (2021). European Journal of Case Reports in Internal Medicine 8(8). | 23 | 7 | 0 | 76.67 |
| Thompson, A. A. and S. Patrawala (2021). BMJ Case Reports 14(1). | 25 | 5 | 0 | 83.33 |
| Toga, A., et al. (2021). JBJS Case Connector 11(1). | 25 | 5 | 0 | 83.33 |
| Watanabe, T., et al. (2021). Yonago Acta Medica 64(3): 318-323. | 23 | 7 | 0 | 76.67 |
| Ahmetgjekaj, I., et al. (2022). Radiology Case Reports 17(9): 3243-3246. | 20 | 9 | 1 | 66.67 |
| Anthony, M. D., et al. (2022). Lymphatic Research and Biology 20(2): 118-124. | 24 | 6 | 0 | 80 |
| Aouad, P., et al. (2022). Child's Nervous System 38(2): 455-460. | 21 | 7 | 2 | 70 |
| Hong, P. Y., et al. (2022). Medicine (United States) 101(50): E32105. | 21 | 7 | 2 | 70 |
| Hyseni, F., et al. (2022). Radiology Case Reports 17(3): 492-495. | 19 | 6 | 5 | 63.33 |
| Krishnan, A., et al. (2022). Surgical Neurology International 13. | 22 | 7 | 1 | 73.33 |
| Maroufi, S. F., et al. (2022). Childs Nervous System 38(4): 695-703. | 25 | 5 | 0 | 83.33 |
| Mbaga, A. C., et al. (2022). Acta Orthopaedica Belgica 88(3): 475-481. | 22 | 8 | 0 | 73.33 |
| Morinaga, Y., et al. (2022). NMC Case Rep J 9: 55-61. | 24 | 5 | 1 | 80 |
| Roy, A., et al. (2022). Advances in Radiation Oncology 7(1). | 23 | 3 | 4 | 76.67 |
| Sattar, A., et al. (2022). BMJ Case Reports 15(11). | 22 | 8 | 0 | 73.33 |
| Suzuki, N., et al. (2022). JRSM Open 13(6). | 17 | 12 | 1 | 56.67 |
| Toivonen, L. A., et al. (2022). Endocrinol Diabetes Metab Case Rep 2022. | 23 | 7 | 0 | 76.67 |
| Yébenes Mayordomo, M., et al. (2022). BMC Medical Genomics 15(1). | 19 | 2 | 9 | 63.33 |
| Zhang, H., et al. (2022). Front Surg 9: 981025. | 28 | 2 | 0 | 93.33 |
| Devaraj, U., et al. (2019). Eurasian Journal of Pulmonology 21(3): 207-211. | 28 | 2 | 0 | 93.33 |
| Pickering, M. E., et al. (2023). Joint Bone Spine 90(1): 105478. | 20 | 4 | 6 | 66.67 |
| Total % Yes | 72.52 |  |  |  |

**Table 3. Clinical Features of GSD.**

| Year | Reference | Gender | Age at onset | Age at diagnosis | Bone involvement | Visceral involvement | Symptoms |
| --- | --- | --- | --- | --- | --- | --- | --- |
| 2013 | Asha et al., 2013. Gerodontology;30(4): 296-302. | F | NR | 60 | CR | NR | Pain |
| 2013 | Barman et al., 2013. NeuroRehabilitation;33(1): 121-126. | M | NR | 3 | CR, SP, UL, LL | NR | Pain, dyspnea, NSI, chylothorax |
| 2013 | Barman et al., 2013. NeuroRehabilitation;33(1): 121-126. | M | 8 | 15 | RI, S, SP | NR | Pain, spinal deformity, NSI |
| 2013 | Chen et al., 2013. Experimental and Therapeutic Medicine;5(4): 1017-1018. | M | 7 | 8 | CR, RI, UL | NR | Dyspnea, chylothorax |
| 2013 | Dong et al., 2013. Experimental and Therapeutic Medicine;5(1): 162-164. | M | 14 | 20 | CR | NR | Pain |
| 2013 | Esmailiejah et al., 2013. Archives of Iranian Medicine;16(11): 686-690. | F | 32 | 32 | SP, LL | Lung | Pain, NSI |
| 2013 | Huang et al., 2013. Respiratory Care;58(11): e144-e148. | M | 28 | 34 | SP, RI | NR | Pain, spinal deformity, dyspnea, NSI, chylothorax |
| 2013 | Kilicoglu et al., 2013. Spine Journal;13(5): e11-e14. | M | 32 | 35 | CR, SP | NR | Pain |
| 2013 | Leite et al., 2013. Pediatric Dermatology;30(3): 374-378. | F | 0 | 7 | SP, LL | NR | Pain, a purple–brown plaque |
| 2013 | Liu et al., 2013. Thoracic Cancer;4(2): 207-211. | M | NR | 32 | RI, SP | NR | Pain, pathological fracture, chylothorax |
| 2013 | Maruani et al., 2013. Pediatric Dermatology;30(3): 391-394. | M | 0 | 6 | LL | NR | Pathological fracture, swelling, a subcutaneous yellowish mass |

| Surgery | | Sirolimus | Interferon | Bisphosphonates | RT | | | PT | | Quality of life | | Imaging evaluation | |  |  |
| --- | --- | --- | --- | --- | --- | --- | --- | --- | --- | --- | --- | --- | --- | --- | --- |
| NO | | NO | NO | NO | NO | | | NO | | NR | | PD | |  |  |
| YES | | NO | NO | YES | NO | | | YES | | SD | | NR | |  |  |
| YES | | NO | NO | YES | YES | | | YES | | PR | | NR | |  |  |
| YES | | NO | NO | NO | YES | | | NO | | SD | | SD | |  |  |
| NR | | NR | NR | NR | NR | | | NR | | NR | | NR | |  |  |
| YES | | NO | NO | YES | NO | | | NO | | PR | | SD | |  |  |
| YES | | NO | YES | NO | YES | | | YES | | NR | | NR | |  |  |
| YES | | NO | NO | NO | NO | | | YES | | SD | | NR | |  |  |
| YES | | NO | YES | YES | NO | | | NO | | PR | | NR | |  |  |
| NO | | NO | YES | NO | YES | | | NO | | PR | | PD | |  |  |
| YES | | NO | NO | YES | NO | | | YES | | PD | | PD | |  |  |
|  | |  |  |  |  | | |  | |  | |  | |  |  |
| Year | Reference | | | | | Gender | Age at onset | | Age at diagnosis | | Bone involvement | | Visceral involvement | | Symptoms |
| 2013 | Morimoto et al., 2013. International Journal of Pediatric Otorhinolaryngology;77(9): 1596-1600. | | | | | F | 11 | | 11 | | CR | | NR | | Pain, dyspnea, CSFL, mild conductive hearing loss |
| 2013 | Noda et al., 2013. General Thoracic and Cardiovascular Surgery;61(6): 356-358. | | | | | M | 15 | | 15 | | SP | | Lung | | Pain, dyspnea, chylothorax |
| 2013 | Ozbayrak et al., 2013. Korean Journal of Radiology;14(6): 946-950. | | | | | M | 38 | | 40 | | UL | | NR | | Pain |
| 2013 | Ravindran et al., 2013. Case Rep Dent;2013: 723583. | | | | | M | 62 | | 62 | | CR | | NR | | Swelling |
| 2013 | Sekharappa et al., 2013. Asian Spine J;7(3): 242-247. | | | | | M | 8 | | 15 | | SP, RI | | NR | | Pain, spinal deformity, dyspnea, NSI |
| 2013 | Sekharappa et al., 2013. Asian Spine J;7(3): 242-247. | | | | | M | 3 | | 23 | | SP, UL | | NR | | Dyspnea, spinal deformity, NSI, chylothorax |
| 2013 | Situma et al., 2013. Journal of Pediatric Surgery;48(1): 239-242. | | | | | M | 0 | | 11 | | SP, LL | | NR | | Pain, dyspnea, chylothorax, a cystic mass |
| 2013 | Stojsic et al., 2013. Journal of Orthopaedic Science;18(1): 186-189. | | | | | M | NR | | 26 | | RI | | NR | | Asymptomatic |
| 2013 | Tavakoli et al., 2013. Trauma Mon;18(1): 41-45. | | | | | F | 51 | | 60 | | UL | | NR | | Pain, pathological fracture |
| 2013 | Tripathy et al., 2013. Orthopaedic Surgery;5(1): 72-76. | | | | | M | 54 | | 54 | | LL | | NR | | Pain, pathological fracture |
| 2013 | Yang et al., 2013. Clin Nucl Med;38(5): 361-364. | | | | | M | 25 | | 31 | | RI, SP | | NR | | Pain, pathological fracture, NSI |
| 2014 | Choi et al., 2014. Pediatr Gastroenterol Hepatol Nutr;17(1): 52-56. | | | | | M | 11 | | 13 | | SP, LL | | Lung | | Pain, chylothorax, melena |

| Surgery | Sirolimus | Interferon | Bisphosphonates | RT | PT | Quality of life | Imaging evaluation |
| --- | --- | --- | --- | --- | --- | --- | --- |
| YES | NO | YES | NO | NO | NO | PR | SD |
| YES | NO | YES | NO | NO | YES | PR | SD |
| NO | NO | NO | NO | YES | NO | PR | SD |
| YES | NO | NO | NO | NO | NO | NR | PD |
| YES | NO | NO | YES | NO | YES | PR | SD |
| YES | NO | NO | NO | NO | YES | SD | SD |
| YES | YES | NO | NO | YES | YES | Dead | Dead |
| NR | NR | NR | NR | NR | NR | NR | NR |
| NR | NR | NR | NR | NR | NR | NR | NR |
| YES | NO | NO | NO | YES | NO | PR | SD |
| NR | NR | NR | NR | NR | NR | NR | NR |
| YES | NO | NO | NO | NO | NO | PR | NR |

| Year | | Reference | | | | Gender | Age at onset | | Age at diagnosis | | Bone involvement | | Visceral involvement | | Symptoms |
| --- | --- | --- | --- | --- | --- | --- | --- | --- | --- | --- | --- | --- | --- | --- | --- |
| 2014 | | Coulter et al., 2014. Clinical Neurology and Neurosurgery;116: 83-86. | | | | F | NR | | 37 | | CR | | NR | | Pain |
| 2014 | | Gem et al., 2014. Acta Orthopaedica Et Traumatologica Turcica;48(4): 467-471. | | | | M | 52 | | 53 | | LL | | NR | | Pain, hyperpigmentation |
| 2014 | | Kakhaki et al., 2014. Iranian Red Crescent Medical Journal;16(11). | | | | M | 48 | | 48 | | RI | | Lung | | Pain, dyspnea, PE |
| 2014 | | Koo et al., 2014. Journal of Rheumatology;41(7): 1430-1432. | | | | F | 30 | | 37 | | SP, LL | | NR | | Pain |
| 2014 | | Posadas et al., 2014. European Review for Medical and Pharmacological Sciences;18(1): 81-83. | | | | F | 15 | | 15 | | CR, SP, LL | | NR | | Epistaxis |
| 2014 | | Scheller et al., 2014. Oral and maxillofacial surgery;18(1): 75-79. | | | | M | 76 | | 76 | | CR | | NR | | Pathological fracture, a exudation of the left external ear |
| 2014 | | Suero et al., 2014. J Neurosurg Spine;21(6): 956-960. | | | | M | NR | | 13 | | SP, RI | | Spleen | | Pain, pathological fracture, chylothorax |
| 2015 | | Alves et al., 2015. Nucl Med Rev Cent East Eur;18(2): 97-101. | | | | M | 44 | | 48 | | LL | | NR | | Pain |
| 2015 | | Baud et al., 2015. BMC research notes;8: 333. | | | | F | 9 | | 15 | | LL | | Spleen | | NR |
| 2015 | | Carbó et al., 2015. European Spine Journal. | | | | M | NR | | 6 | | SP, LL | | NR | | Pain, a cystic mass |
| 2015 | | Davalos et al., 2015. Radiology Case Reports;10(2). | | | | F | NR | | 9 | | RI, SP, UL, LL | | Spleen | | Pain, pathological fracture, dyspnea, chylothorax, a cystic mass |
| Surgery | | Sirolimus | Interferon | Bisphosphonates | RT | | PT | | Quality of life | | Imaging evaluation | |  |  |  |
| NO | | NO | NO | YES | NO | | NO | | NR | | SD | |  |  |  |
| NO | | NO | NO | YES | NO | | NO | | PR | | SD | |  |  |  |
| YES | | NO | NO | YES | YES | | NO | | PR | | SD | |  |  |  |
| NO | | NO | NO | YES | NO | | NO | | SD | | NR | |  |  |  |
| NO | | NO | NO | NO | NO | | NO | | SD | | NR | |  |  |  |
| YES | | NO | NO | NO | NO | | NO | | NR | | SD | |  |  |  |
| YES | | YES | YES | YES | NO | | NO | | PR | | NR | |  |  |  |
| NR | | NR | NR | NR | NR | | NR | | NR | | NR | |  |  |  |
| NO | | NO | NO | NO | NO | | NO | | PR | | SD | |  |  |  |
| YES | | NO | YES | YES | YES | | YES | | PR | | SD | |  |  |  |
| NR | | NR | NR | NR | NR | | NR | | NR | | NR | |  |  |  |

| Year | Reference | | | | | Gender | Age at onset | | Age at diagnosis | | Bone involvement | | Visceral involvement | | Symptoms |
| --- | --- | --- | --- | --- | --- | --- | --- | --- | --- | --- | --- | --- | --- | --- | --- |
| 2015 | Duraisamy et al., 2015. J Clin Diagn Res;9(11): Zd25-27. | | | | | F | 29 | | 29 | | CR | | NR | | Difficulty in opening mouth |
| 2015 | ndler et al., 2015. Lymphatic Research and Biology;13(1): 62-65. | | | | | F | 50 | | 59 | | CR, LL | | Lung | | Dyspnea, PE, peritoneal effusion, pericardial effusion |
| 2015 | Gulati et al., 2015. J Maxillofac Oral Surg;14(3): 548-557. | | | | | M | 18 | | 18 | | CR | | NR | | Pain, swelling |
| 2015 | Karim et al., 2015. American journal of orthopedics;44(11): E473-E477. | | | | | M | 27 | | 27 | | SP, LL | | NR | | Pain |
| 2015 | Kim et al., 2015. Korean J Thorac Cardiovasc Surg;48(1): 90-94. | | | | | M | 15 | | 16 | | S | | NR | | Pain, a mass |
| 2015 | Kim et al., 2015. Journal of Oral and Maxillofacial Surgery;73(12): 2352-2360. | | | | | M | 12 | | 12 | | CR, SP | | NR | | Pain, dyspnea, PE |
| 2015 | Kohno et al., 2015. NMC Case Rep J;2(2): 80-84. | | | | | M | 27 | | 27 | | SP | | NR | | Pain, pathological fracture, NSI |
| 2015 | Kunjur et al., 2015. Journal of Craniofacial Surgery;26(4): E302-E305. | | | | | M | NR | | 45 | | CR | | NR | | Pain |
| 2015 | Ohla et al., 2015. BMC surgery;15: 24. | | | | | M | 22 | | 25 | | CR | | NR | | Pain |
| 2015 | Pn et al., 2015. J Orthop Case Rep;5(4): 30-33. | | | | | M | NR | | 11 | | SP, RI, UL | | NR | | Pain, pathological fracture |
| 2015 | Rössler et al., 2015. Journal of Pediatric Hematology/Oncology;37(8): e481-e485. | | | | | M | NR | | 14 | | SP, LL | | NR | | Pain, swelling |
| 2015 | Sá et al., 2015. Rev Bras Ortop;50(2): 239-242. | | | | | M | 43 | | 48 | | LL | | NR | | Pain |
| Surgery | | Sirolimus | Interferon | Bisphosphonates | RT | | | PT | | Quality of life | | Imaging evaluation | |  |  |
| YES | | NO | NO | NO | NO | | | NO | | PR | | SD | |  |  |
| YES | | NO | NO | NO | NO | | | NO | | Dead | | Dead | |  |  |
| YES | | NO | NO | NO | NO | | | NO | | PR | | SD | |  |  |
| YES | | YES | NO | YES | YES | | | NO | | PR | | NR | |  |  |
| YES | | NO | YES | NO | YES | | | NO | | PR | | SD | |  |  |
| NO | | NO | YES | YES | YES | | | NO | | Dead | | Dead | |  |  |
| YES | | NO | NO | YES | YES | | | YES | | PR | | SD | |  |  |
| YES | | NO | NO | NO | NO | | | NO | | PR | | NR | |  |  |
| YES | | NO | NO | NO | NO | | | NO | | NR | | SD | |  |  |
| NO | | NO | NO | YES | NO | | | YES | | PD | | PD | |  |  |
| NO | | NO | NO | NO | NO | | | YES | | PR | | NR | |  |  |
| NR | | NR | NR | NR | NR | | | NR | | NR | | NR | |  |  |

| Year | Reference | Gender | Age at onset | Age at diagnosis | Bone involvement | Visceral involvement | Symptoms |
| --- | --- | --- | --- | --- | --- | --- | --- |
| 2015 | Shi et al., 2015. Skeletal Radiology;44(7): 1033-1038. | M | 26 | 26 | UL | NR | Pain, swelling |
| 2015 | Yerganyan et al., 2015. Journal of Bone Oncology;4(2): 42-46. | M | 28 | 28 | LL | NR | Pain, pathological fracture, limping |
| 2015 | Zhao et al., 2015. The Journal of craniofacial surgery;26(2): e160-e162. | M | NR | 46 | CR | NR | Pain, pathological fracture, swelling |
| 2016 | Amirjamshidi et al., 2016. British Journal of Neurosurgery;30(6): 687-688. | M | 60 | 62 | CR | NR | Pain |
| 2016 | Chan et al., 2016. Malays Orthop J;10(3): 42-45. | F | 45 | 46 | LL | NR | Pain |
| 2016 | Cramer et al., 2016. Journal of Pediatric Hematology/Oncology;38(3): e129-e132. | M | 18 | 18 | RI | NR | Pain, pathological fracture, dyspnea, chylothorax |
| 2016 | Demir et al., 2016. Spine Journal;16(1): e19-e21. | M | NR | 35 | SP, RI, UL | NR | Pain |
| 2016 | Ellati et al., 2016. European Review for Medical and Pharmacological Sciences;20(3): 426-432. | M | 18 | 18 | UL | NR | Pain, swelling |
| 2016 | Evrenos et al., 2016. J Maxillofac Oral Surg;15(Suppl 2): 335-338. | M | 19 | 21 | CR, LL | NR | Dyspnea, chylothorax, pericardial effusion |
| 2016 | Ganal-Antonio et al., 2016. Spine J;16(2): e67-75. | F | 5 | 26 | SP, UL, LL | NR | Pain, pathological fracture, swelling |
| 2016 | García et al., 2016. Colombia Medica;47(4): 213-216. | F | 41 | 43 | RI | NR | Pain, dyspnea, PE, abdominal mass |
| 2016 | Gataa et al., 2016. Journal of Oral and Maxillofacial Surgery;74(9): 1774-1782. | F | 13 | 15 | CR | NR | Pain, swelling |

| Surgery | Sirolimus | Interferon | Bisphosphonates | RT | PT | Quality of life | Imaging evaluation |
| --- | --- | --- | --- | --- | --- | --- | --- |
| YES | NO | NO | NO | NO | NO | PR | PR |
| NO | NO | NO | YES | YES | NO | PR | PR |
| YES | NO | NO | NO | NO | NO | SD | PD |
| YES | NO | YES | YES | NO | NO | PR | SD |
| YES | NO | NO | NO | NO | NO | PR | SD |
| YES | YES | YES | YES | NO | NO | PR | SD |
| YES | NO | NO | NO | NO | NO | NR | NR |
| YES | NO | NO | YES | YES | NO | PR | SD |
| YES | NO | NO | YES | NO | NO | SD | NR |
| YES | NO | NO | YES | NO | YES | Dead | Dead |
| YES | YES | NO | NO | NO | NO | PR | SD |
| YES | NO | NO | NO | NO | NO | PR | SD |

| Year | Reference | Gender | Age at onset | Age at diagnosis | Bone involvement | Visceral involvement | Symptoms |
| --- | --- | --- | --- | --- | --- | --- | --- |
| 2016 | Mukulchotrani et al., 2016. Pravara Medical Review;8(3): 26-28. | F | 30 | NR | UL | NR | Pain, swelling |
| 2016 | Nozawa et al., 2016. Pediatric Blood and Cancer;63(5): 931-934. | M | NR | 6 | CR | NR | NSI, CSFL, meningitis, hearing loss |
| 2016 | Tolis et al., 2016. Journal of Musculoskeletal Neuronal Interactions;16(1): 79-82. | F | 22 | 22 | LL | NR | Pain, swelling |
| 2016 | Yamagishi et al., 2016. Journal of Pediatric Orthopaedics;Part B 25(1): 48-53. | M | 10 | 12 | LL | NR | Pain, pathological fracture, swelling |
| 2016 | Zakhary et al.,2016. British Journal of Oral and Maxillofacial Surgery;54(7): 845-846. | F | NR | 54 | CR | NR | Loss of tooth |
| 2017 | Bargagli et al., 2017. AJP Reports;7(4): E226-E229. | F | 32 | 32 | SP, S | Lung, spleen | Pain, dyspnea, chylothorax, chylous ascites |
| 2017 | Bocchialini et al., 2017. International Journal of Surgery Case Reports;34: 110-114. | F | NR | 14 | CR, SP, LL | NR | Mandibular hypoplasia |
| 2017 | Jose et al., 2017. Clinical Reviews in Allergy & Immunology;52(1): 125-132. | F | 6 | 8 | CR | NR | Pain, swelling, purple scar |
| 2017 | Kato et al., 2017. Japanese Journal of Radiology;35(10): 606-612. | F | 2 | NR | SP, LL | NR | Asymptomatic |
| 2017 | Kim et al., 2017. Indian J Orthop;51(2): 217-221. | M | 18 | 19 | UL | NR | Pain, pathological fracture, swelling |
| 2017 | Klein et al., 2017. Journal of Pediatrics;191: 277-277.e271. | F | NR | 8 | UL | NR | Pain, swelling |
| 2017 | Liu et al., 2017. Medicine (Baltimore);96(42): e8184. | M | 28 | 29 | CR | NR | Pain, loosening of tooth |

| Surgery | Sirolimus | Interferon | Bisphosphonates | RT | PT | Quality of life | Imaging evaluation |
| --- | --- | --- | --- | --- | --- | --- | --- |
| NR | NR | NR | NR | NR | NR | NR | NR |
| YES | YES | YES | NO | NO | NO | PR | SD |
| NO | NO | NO | YES | YES | NO | PD | PD |
| YES | NO | NO | NO | NO | NO | PR | SD |
| NO | NO | NO | NO | NO | NO | NR | NR |
| YES | NO | NO | NO | NO | NO | SD | NR |
| NO | NO | NO | YES | NO | NO | NR | NR |
| NO | NO | NO | NO | NO | YES | NR | NR |
| NR | NR | NR | NR | NR | NR | NR | NR |
| YES | NO | NO | YES | YES | NO | NR | SD |
| NO | NO | NO | YES | NO | YES | PR | SD |
| NO | NO | NO | NO | NO | NO | NR | NR |

| Year | Reference | Gender | Age at onset | Age at diagnosis | Bone involvement | Visceral involvement | Symptoms |
| --- | --- | --- | --- | --- | --- | --- | --- |
| 2017 | Mohapatra et al., 2017. The Journal of clinical pediatric dentistry;41(6): 472-477. | F | 7 | 7 | CR | NR | Facial asymmetry |
| 2017 | Naqvi et al., 2017. J Surg Case Rep;2017(2): rjx025. | F | 25 | 25 | CR | NR | Pain, NSI, CSFL, meningitis |
| 2017 | Páez Codeso et al., 2017. Archivos De Bronconeumologia;53(11): 640. | M | NR | 14 | UL | NR | Pain, pathological fracture, chylothorax |
| 2017 | Park et al., 2017. Clinical Nuclear Medicine;42(10): 779-781. | M | 13 | 13 | CR | NR | Pain, loosening of tooth |
| 2017 | Renacci, R. M. and R. J. Bartolotta, 2017. Clinical Imaging;41: 83-85. | M | NR | 30 | SP, LL | NR | Pain, hematoma |
| 2017 | Sinha et al., 2017. Iran J Pathol;12(3): 301-306. | M | 28 | 38 | CR | NR | Pathological fracture, loss of tooth |
| 2017 | Srivastava et al., 2017. Indian J Orthop;51(1): 107-114. | M | 17 | 17 | SP, RI | NR | Spinal deformity, NSI |
| 2017 | Tateda et al., 2017. Tohoku Journal of Experimental Medicine;241(4): 249-254. | M | NR | 15 | SP | NR | Pain, chylothorax |
| 2017 | Zhao et al., 2017. Rheumatology;56(8): 1311-1311. | F | 40 | 46 | CR | NR | Skull indentation |
| 2018 | Bruyère et al., 2018. Journal of Solid Tumors;8(2): 20-24. | F | 54 | 55 | UL | NR | Pain |
| 2018 | Costa et al., 2018. Journal of Clinical and Diagnostic Research;12(5): PD17-PD19. | M | NR | 10 | CR, SP | NR | Pain, NSI, meningitis |
| 2018 | Dsouza et al., 2018. Journal of Anaesthesiology Clinical Pharmacology;34(1): 132-133. | M | 16 | 16 | SP, RI | NR | Spinal deformity, NSI |

| Surgery | Sirolimus | Interferon | Bisphosphonates | RT | PT | Quality of life | Imaging evaluation |
| --- | --- | --- | --- | --- | --- | --- | --- |
| NO | NO | NO | NO | YES | NO | NR | SD |
| YES | NO | NO | NO | NO | NO | SD | NR |
| YES | NO | YES | YES | NO | NO | SD | SD |
| NO | NO | NO | YES | NO | NO | NR | PD |
| YES | NO | NO | YES | YES | NO | PR | SD |
| NR | NR | NR | NR | NR | NR | NR | NR |
| YES | NO | NO | YES | YES | NO | PR | SD |
| YES | NO | YES | YES | YES | YES | PR | SD |
| NO | NO | YES | YES | YES | NO | SD | SD |
| NO | NO | NO | NO | YES | NO | NR | NR |
| YES | NO | YES | YES | NO | NO | Dead | Dead |
| YES | NO | NO | NO | NO | NO | NR | NR |

| Year | Reference | Gender | Age at onset | Age at diagnosis | Bone involvement | Visceral involvement | Symptoms |
| --- | --- | --- | --- | --- | --- | --- | --- |
| 2018 | Ganau et al., 2018. Hellenic Journal of Nuclear Medicine;21(3): 198-201. | F | 36 | 46 | CR | NR | Pain, NSI |
| 2018 | Hurtado et al., 2018. Revista De Osteoporosis Y Metabolismo Mineral;10(2): 96-97. | M | 48 | 50 | LL | NR | Pain |
| 2018 | Illeez et al., 2018. Orthopade;47(12): 1032-1035. | F | 53 | 55 | LL | NR | Pain, swelling |
| 2018 | Jaccard et al., 2018. Surgical Neurology International;9(1). | F | 13 | 23 | RI | Lung | Pathological fracture, NSI, bloody pleural effusion |
| 2018 | Jatuworapruk et al., 2018. International Journal of Rheumatic Diseases;21(7): 1458-1462. | M | 19 | 21 | RI, S | NR | Pain, dyspnea, chylothorax, chylous ascites, pericardial effusion |
| 2018 | Kotaru et al., 2018. Journal of Bronchology and Interventional Pulmonology;25(4): 340-342. | F | NR | 36 | S, RI, UL | NR | Dyspnea, chylothorax |
| 2018 | Li et al., 2018. Orthopaedic Surgery;10(3): 276-280. | M | 26 | 26 | RI, UL | NR | Pain, PE |
| 2018 | Liu et al., 2018. Medicine;97(29). | M | 24 | 31 | SP, LL | Spleen | Pain, NSI, chylothorax, chylous ascites |
| 2018 | Liu et al., 2018. QJM;111(12): 911-912. | F | 13 | 29 | S, SP, UL | NR | Pain, chylothorax |
| 2018 | Liu et al., 2018. Chin Med J;131(13): 1628-1629. | F | NR | 27 | LL | Spleen | Pain, chylothorax, chylous ascites |
| 2018 | Lova et al., 2018. Radiology Case Reports;13(1): 96-100. | M | 25 | 25 | CR | NR | Pain, swelling |
| 2018 | Maillot et al., 2018. European spine journal;27(9): 2285-2290. | F | NR | 20 | SP | NR | Pain, pathological fracture, spinal deformity, NSI, chylothorax |

| Surgery | Sirolimus | Interferon | Bisphosphonates | RT | PT | Quality of life | Imaging evaluation |
| --- | --- | --- | --- | --- | --- | --- | --- |
| YES | NO | NO | YES | NO | NO | PR | NR |
| NR | NR | NR | NR | NR | NR | NR | NR |
| NO | NO | NO | YES | NO | YES | PR | PR |
| YES | NO | NO | NO | YES | YES | PR | NR |
| YES | NO | YES | YES | YES | NO | Dead | Dead |
| YES | NO | NO | NO | NO | NO | NR | NR |
| YES | NO | NO | YES | YES | NO | PR | SD |
| YES | NO | YES | YES | NO | NO | PR | NR |
| NO | NO | YES | YES | NO | NO | PR | NR |
| YES | NO | YES | YES | NO | NO | SD | SD |
| NO | NO | NO | YES | NO | NO | NR | NR |
| YES | NO | NO | YES | NO | YES | PR | NR |

| Year | Reference | Gender | Age at onset | Age at diagnosis | Bone involvement | Visceral involvement | Symptoms |
| --- | --- | --- | --- | --- | --- | --- | --- |
| 2018 | Mo et al., 2018. JBJS Case Connect;8(3): e70. | M | 11 | 14 | RI, SP | NR | Spinal deformity |
| 2018 | Noman et al., 2018. Journal of the College of Physicians and Surgeons;28(9): S164-S165. | F | 17 | 17 | UL | NR | Pain, pathological fracture |
| 2018 | Reipschläger et al., 2018. JAAD Case Reports;4(5): 458-461. | M | 0 | 1.5 | UL | NR | Pain, pathological fracture, dilated cutaneous venules |
| 2018 | Soh et al., 2018. Oral Radiology;34(2): 179-184. | M | NR | 15 | CR | NR | Lower jaw deformity, loosening of tooth |
| 2018 | Stevens et al., 2018. BMJ Case Reports;2018. | F | NR | 45 | UL | NR | Pain, pathological fracture, swelling |
| 2018 | Stroh et al., 2018. Journal of Neuro-Ophthalmology;38(1): 70-74. | F | 66 | 66 | CR, SP, RI, LL | NR | Pain, NSI |
| 2018 | Tasis et al., 2018. Case Rep Pulmonol;2018: 2406496. | F | 24 | 35 | SP, UL, LL | Spleen | Pain, cystic mass |
| 2018 | Yoshimoto et al., 2018. Journal of Neurosurgery: Pediatrics;22(5): 508-512. | F | 2 | 13 | LL | NR | Pathological fracture, CSFL |
| 2019 | Ali et al., 2019. Cureus;11(6): e4832. | F | 18 | 18 | UL | NR | Pain |
| 2019 | Andrade et al., 2019. Radiologia brasileira;52(2): 131-133. | M | 33 | 34 | RI, SP, UL, LL | NR | Pain, swelling |
| 2019 | Barbagli et al., 2019. Interdisciplinary Neurosurgery: Advanced Techniques and Case Management;17: 79-83. | M | NR | 29 | SP | NR | Pathological fracture, NSI, cellulitis, chylothorax |

| Surgery | Sirolimus | Interferon | Bisphosphonates | RT | PT | Quality of life | Imaging evaluation |
| --- | --- | --- | --- | --- | --- | --- | --- |
| YES | YES | NO | YES | NO | NO | PR | PR |
| NR | NR | NR | NR | NR | NR | NR | NR |
| YES | NO | NO | NO | NO | NO | PR | PR |
| YES | NO | YES | NO | NO | NO | NR | NR |
| NR | NR | NR | NR | NR | NR | NR | NR |
| YES | NO | NO | YES | NO | NO | NR | NR |
| YES | NO | NO | NO | NO | NO | NR | NR |
| YES | YES | YES | NO | NO | NO | PR | NR |
| NR | NR | NR | NR | NR | NR | NR | NR |
| NR | NR | NR | NR | NR | NR | NR | NR |
| YES | YES | NO | NO | NO | NO | Dead | Dead |

| Year | | Reference | | | | | Gender | | Age at onset | | | Age at diagnosis | | | Bone involvement | | | Visceral involvement | | | Symptoms | |
| --- | --- | --- | --- | --- | --- | --- | --- | --- | --- | --- | --- | --- | --- | --- | --- | --- | --- | --- | --- | --- | --- | --- |
| 2019 | | Du et al., 2019. Quantitative Imaging in Medicine and Surgery;9(4): 565-578. | | | | | M | | NR | | | 5 | | | SP | | | NR | | | Pain, pathological fracture, spinal deformity | |
| 2019 | | Du et al., 2019. Quantitative Imaging in Medicine and Surgery;9(4): 565-578. | | | | | F | | NR | | | 2 | | | SP, RI, LL | | | NR | | | Pain, pathological fracture, spinal deformity, NSI | |
| 2019 | | Du et al., 2019. Quantitative Imaging in Medicine and Surgery;9(4): 565-578. | | | | | F | | NR | | | 11 | | | SP, RI, S | | | NR | | | Pain, pathological fracture, spinal deformity, NSI | |
| 2019 | | Du et al., 2019. Quantitative Imaging in Medicine and Surgery;9(4): 565-578. | | | | | M | | NR | | | 12 | | | SP, RI | | | NR | | | Pathological fracture, spinal deformity | |
| 2019 | | Du et al., 2019. Quantitative Imaging in Medicine and Surgery;9(4): 565-578. | | | | | F | | NR | | | 39 | | | SP | | | NR | | | Pain, pathological fracture, spinal deformity, NSI | |
| 2019 | | Du et al., 2019. Quantitative Imaging in Medicine and Surgery;9(4): 565-578. | | | | | F | | NR | | | 43 | | | SP | | | NR | | | Pain, pathological fracture, spinal deformity, NSI | |
| 2019 | | Du et al., 2019. Quantitative Imaging in Medicine and Surgery;9(4): 565-578. | | | | | M | | NR | | | 12 | | | SP, RI | | | NR | | | Pathological fracture, spinal deformity | |
| 2019 | | Du et al., 2019. Quantitative Imaging in Medicine and Surgery;9(4): 565-578. | | | | | F | | NR | | | 11 | | | SP, RI, S, UL, LL | | | NR | | | Pathological fracture, spinal deformity | |
| 2019 | | Du et al., 2019. Quantitative Imaging in Medicine and Surgery;9(4): 565-578. | | | | | M | | NR | | | 10 | | | SP | | | NR | | | Pathological fracture, spinal deformity | |
| 2019 | | Du et al., 2019. Quantitative Imaging in Medicine and Surgery;9(4): 565-578. | | | | | M | | NR | | | 12 | | | SP, RI, UL | | | NR | | | Pain, pathological fracture, spinal deformity, NSI | |
| 2019 | | Du et al., 2019. Quantitative Imaging in Medicine and Surgery;9(4): 565-578. | | | | | F | | NR | | | 10 | | | SP, RI | | | NR | | | Pathological fracture, spinal deformity | |
| 2019 | | Feng et al., 2019. BMC Musculoskeletal Disorders;20(1). | | | | | F | | 52 | | | 52 | | | UL | | | NR | | | Pain, swelling, NSI | |
| Surgery | | | Sirolimus | Interferon | Bisphosphonates | RT | | | | PT | | | Quality of life | | | Imaging evaluation | | |  |  |  |  |
| NO | | | NO | NO | YES | NO | | | | YES | | | NR | | | NR | | |  |  |  |  |
| YES | | | NO | NO | YES | NO | | | | NO | | | PD | | | PD | | |  |  |  |  |
| NO | | | NO | NO | YES | NO | | | | NO | | | PD | | | PR | | |  |  |  |  |
| YES | | | NO | NO | YES | NO | | | | YES | | | PR | | | PR | | |  |  |  |  |
| YES | | | NO | NO | YES | NO | | | | NO | | | PR | | | PR | | |  |  |  |  |
| NO | | | NO | NO | YES | NO | | | | NO | | | PD | | | SD | | |  |  |  |  |
| NO | | | NO | NO | YES | NO | | | | NO | | | PD | | | PD | | |  |  |  |  |
| NO | | | NO | NO | YES | NO | | | | YES | | | SD | | | PR | | |  |  |  |  |
| YES | | | NO | NO | YES | NO | | | | NO | | | PR | | | SD | | |  |  |  |  |
| YES | | | NO | NO | YES | NO | | | | NO | | | PD | | | SD | | |  |  |  |  |
| NO | | | NO | NO | YES | NO | | | | YES | | | SD | | | PR | | |  |  |  |  |
| YES | | | NO | NO | NO | NO | | | | NO | | | PR | | | SD | | |  |  |  |  |
| Year | | Reference | | | | | Gender | | Age at onset | | | Age at diagnosis | | | Bone involvement | | | Visceral involvement | | | Symptoms | |
| 2019 | | Ferreiro et al., 2019. Pulmonology;25(3): 195-197. | | | | | M | | 37 | | | 37 | | | SP, RI, LL | | | NR | | | Dyspnea, chylothorax | |
| 2019 | | Hickmann et al., 2019. Rheumatology;58(7): 1314-1315. | | | | | M | | NR | | | 39 | | | CR, SP, S, RI, UL, LL | | | NR | | | Pain, dyspnea | |
| 2019 | | Jiang et al., 2019. Medicine;98(14). | | | | | F | | 5 | | | 15 | | | SP, RI, UL, LL | | | NR | | | Pain, dyspnea, chylothorax, pericardial effusion | |
| 2019 | | Kaissi et al., 2019. Medicines;6(2). | | | | | M | | 3 | | | 7 | | | SP, LL | | | NR | | | Pain, spinal deformity | |
| 2019 | | Kaissi et al., 2019. Medicines;6(2). | | | | | F | | 6 | | | 13 | | | UL, LL | | | NR | | | Pathological fracture | |
| 2019 | | Kim et al., 2019. World Neurosurgery;129: 277-281. | | | | | M | | 21 | | | 22 | | | SP, RI | | | NR | | | Pain, pathological fracture, dyspnea, chylothorax | |
| 2019 | | Koto et al., 2019. Molecular and Clinical Oncology;11(6): 551-556. | | | | | M | | 77 | | | 77 | | | RI, SP | | | Liver, spleen | | | Dyspnea, NSI, bloody pleural effusion | |
| 2019 | | Matsumoto et al., 2019. JBJS Case Connector;9(2). | | | | | M | | 41 | | | 47 | | | UL | | | NR | | | NR | |
| 2019 | | Matsumoto et al., 2019. JBJS Case Connector;9(2). | | | | | F | | 70 | | | 72 | | | UL | | | NR | | | Swelling | |
| 2019 | | Nozawa et al., 2019. Internal Medicine;58(13): 1929-1933. | | | | | M | | 27 | | | 33 | | | CR | | | NR | | | NSI | |
| 2019 | | Schneider et al., 2019. Deutsches Arzteblatt International;116(29-30): 507. | | | | | F | | 28 | | | 29 | | | LL | | | NR | | | Pain | |
| 2019 | | Simon et al., 2019. Int J Pediatr Otorhinolaryngol;124: 152-156. | | | | | NR | | 3 | | | NR | | | CR, SP | | | NR | | | Chiari I malformation, CSFL, meningitis, hearing loss | |
| Surgery | | | Sirolimus | Interferon | Bisphosphonates | RT | | | | PT | | | Quality of life | | | Imaging evaluation | | |  |  |  |  |
| YES | | | YES | NO | NO | NO | | | | NO | | | PR | | | SD | | |  |  |  |  |
| YES | | | NO | NO | YES | NO | | | | NO | | | PR | | | NR | | |  |  |  |  |
| YES | | | NO | NO | YES | NO | | | | NO | | | PR | | | NR | | |  |  |  |  |
| NO | | | NO | NO | NO | NO | | | | YES | | | SD | | | SD | | |  |  |  |  |
| YES | | | NO | NO | NO | NO | | | | NO | | | NR | | | NR | | |  |  |  |  |
| YES | | | NO | NO | NO | NO | | | | NO | | | PR | | | NR | | |  |  |  |  |
| YES | | | NO | NO | YES | YES | | | | NO | | | Dead | | | Dead | | |  |  |  |  |
| NO | | | NO | NO | YES | NO | | | | NO | | | NR | | | SD | | |  |  |  |  |
| NO | | | NO | NO | NO | NO | | | | NO | | | NR | | | SD | | |  |  |  |  |
| NO | | | YES | NO | YES | YES | | | | NO | | | Dead | | | Dead | | |  |  |  |  |
| NO | | | NO | NO | YES | NO | | | | NO | | | PR | | | SD | | |  |  |  |  |
| YES | | | NO | NO | NO | NO | | | | NO | | | NR | | | NR | | |  |  |  |  |
| Year | | Reference | | | | | Gender | | Age at onset | | | Age at diagnosis | | | Bone involvement | | | Visceral involvement | | | Symptoms | |
| 2019 | | Simon et al., 2019. Int J Pediatr Otorhinolaryngol;124: 152-156. | | | | | NR | | 3 | | | NR | | | CR | | | NR | | | Chiari I malformation, CSFL, meningitis | |
| 2019 | | Simon et al., 2019. Int J Pediatr Otorhinolaryngol;124: 152-156. | | | | | NR | | 1 | | | NR | | | CR | | | NR | | | Chiari I malformation, CSFL, meningitis, hearing loss | |
| 2019 | | Simon et al., 2019. Int J Pediatr Otorhinolaryngol;124: 152-156. | | | | | NR | | 10 | | | NR | | | CR, SP | | | NR | | | Chiari I malformation | |
| 2019 | | Simon et al., 2019. Int J Pediatr Otorhinolaryngol;124: 152-156. | | | | | NR | | 0 | | | NR | | | CR | | | NR | | | CSFL, meningitis | |
| 2019 | | Simon et al., 2019. Int J Pediatr Otorhinolaryngol;124: 152-156. | | | | | NR | | 5 | | | NR | | | CR, SP | | | NR | | | Chiari I malformation | |
| 2019 | | Tena-Sanabria et al., 2019. BMC Pediatrics;19(1). | | | | | M | | 7 | | | 12 | | | LL | | | NR | | | Pain | |
| 2019 | | Tena-Sanabria et al., 2019. BMC Pediatrics;19(1). | | | | | M | | 0.58 | | | 2.58 | | | SP | | | NR | | | Pain, Chiari I malformation | |
| 2019 | | Wang et al., 2019. BMC Musculoskeletal Disorders;20(1). | | | | | F | | 14 | | | 14 | | | SP, RI | | | NR | | | Pain, pathological fracture, spinal deformity, dyspnea, chylothorax | |
| 2019 | | Zheng et al., 2019. BMC Musculoskeletal Disorders;21(1). | | | | | M | | 40 | | | 40 | | | LL | | | NR | | | Pain, swelling | |
| 2019 | | Devaraj et al., 2019. Eurasian Journal of Pulmonology;21(3): 207-211. | | | | | M | | 24 | | | 25 | | | SP, RI | | | Lung | | | Pain, dyspnea, PE | |
| 2020 | | Al Baroudi et al., 2020. Am J Respir Crit Care Med;202(3): 451-452. | | | | | M | | NR | | | 15 | | | RI | | | NR | | | Dyspnea, PE | |
| 2020 | | Chang et al., 2020. Experimental and Therapeutic Medicine;19(6): 3851-3855. | | | | | M | | NR | | | 22 | | | SP | | | NR | | | Pain, dyspnea, chylothorax | |
| Surgery | | | Sirolimus | Interferon | Bisphosphonates | RT | | | | PT | | | Quality of life | | | Imaging evaluation | | |  |  |  |  |
| YES | | | NO | NO | NO | NO | | | | NO | | | NR | | | NR | | |  |  |  |  |
| YES | | | NO | NO | NO | NO | | | | NO | | | NR | | | NR | | |  |  |  |  |
| YES | | | NO | NO | NO | NO | | | | NO | | | Dead | | | Dead | | |  |  |  |  |
| YES | | | NO | NO | NO | NO | | | | NO | | | NR | | | NR | | |  |  |  |  |
| YES | | | NO | NO | NO | NO | | | | NO | | | Dead | | | Dead | | |  |  |  |  |
| YES | | | NO | NO | NO | NO | | | | NO | | | SD | | | PD | | |  |  |  |  |
| YES | | | NO | YES | YES | YES | | | | NO | | | Dead | | | Dead | | |  |  |  |  |
| YES | | | NO | NO | YES | NO | | | | YES | | | PR | | | NR | | |  |  |  |  |
| YES | | | NO | NO | YES | NO | | | | NO | | | PR | | | SD | | |  |  |  |  |
| YES | | | NO | YES | NO | NO | | | | YES | | | PR | | | PR | | |  |  |  |  |
| NO | | | YES | NO | YES | NO | | | | NO | | | NR | | | NR | | |  |  |  |  |
| YES | | | NO | NO | YES | YES | | | | NO | | | PR | | | SD | | |  |  |  |  |
| Year | | Reference | | | | | Gender | | Age at onset | | | Age at diagnosis | | | Bone involvement | | | Visceral involvement | | | Symptoms | |
| 2020 | | Elera-Fitzcarrald et al., 2020. Jcr-Journal of Clinical Rheumatology;26(5): E135-E136. | | | | | M | | 39 | | | 43 | | | RI | | | NR | | | Pain, swelling, PE | |
| 2020 | | Hosoya et al., 2020. Journal of Otolaryngology - Head and Neck Surgery;49(1). | | | | | M | | 2 | | | 25 | | | CR | | | NR | | | Pain, NSI, CSFL, meningitis, otorrhea | |
| 2020 | | Hou et al., 2020. Medicine;99(39): E22414. | | | | | F | | NR | | | NR | | | SP, RI | | | Lung | | | Pain, dyspnea, PE, pericardial effusion | |
| 2020 | | Hou et al., 2020. Medicine;99(39): E22414. | | | | | M | | NR | | | NR | | | SP, LL | | | NR | | | Pain, peritoneal effusion | |
| 2020 | | Hou et al., 2020. Medicine;99(39): E22414. | | | | | M | | NR | | | NR | | | LL | | | NR | | | Pain, swelling | |
| 2020 | | Jagtap et al., 2020. Oral Radiol;36(4): 389-394. | | | | | M | | 3 | | | 5 | | | CR | | | NR | | | Pain, pathological fracture, swelling, loss of tooth, ulceration | |
| 2020 | | Jha, V. and A. Jha, 2020. BMJ Case Reports;13(2). | | | | | M | | 0.58 | | | 2.17 | | | LL | | | NR | | | Dyspnea, chylothorax, swelling, hyperpigmentation | |
| 2020 | | Liang et al., 2020. BMC Musculoskeletal Disorders;21(1). | | | | | M | | 0 | | | 1 | | | UL, LL | | | NR | | | Pain, pathological fracture, swelling, dyspnea, PE, cystic mass | |
| 2020 | | Ogawa et al., 2020. J Dermatol;47(11): e388-e390. | | | | | F | | NR | | | 9 | | | LL | | | NR | | | Pathological fracture, spinal deformity, swelling, PE, red-to-brown verrucous papules | |
| 2020 | | Rahman et al., 2020. Journal of Taibah University Medical Sciences;15(2): 160-165. | | | | | F | | 61 | | | 61 | | | CR | | | NR | | | Pain, loosening of tooth | |
| 2020 | | Schneider et al., 2020. Acta Orthopaedica;91(2): 209-214. | | | | | M | | 5 | | | 5 | | | CR, SP, RI, UL, LL | | | NR | | | Pain, swelling, chylothorax | |
| 2020 | | Schneider et al., 2020. Acta Orthopaedica;91(2): 209-214. | | | | | F | | 27 | | | 29 | | | LL | | | NR | | | Pain | |
| Surgery | | | Sirolimus | Interferon | Bisphosphonates | RT | | | | PT | | | Quality of life | | | Imaging evaluation | | |  |  |  |  |
| YES | | | NO | NO | YES | NO | | | | NO | | | PR | | | SD | | |  |  |  |  |
| YES | | | NO | NO | NO | NO | | | | NO | | | PR | | | SD | | |  |  |  |  |
| NR | | | NR | NR | NR | NR | | | | NR | | | NR | | | NR | | |  |  |  |  |
| NR | | | NR | NR | NR | NR | | | | NR | | | NR | | | NR | | |  |  |  |  |
| NR | | | NR | NR | NR | NR | | | | NR | | | NR | | | NR | | |  |  |  |  |
| YES | | | YES | YES | YES | NO | | | | NO | | | NR | | | PD | | |  |  |  |  |
| YES | | | NO | NO | NO | YES | | | | NO | | | PR | | | NR | | |  |  |  |  |
| YES | | | YES | NO | NO | NO | | | | NO | | | PR | | | SD | | |  |  |  |  |
| NO | | | YES | NO | NO | NO | | | | NO | | | PD | | | NR | | |  |  |  |  |
| YES | | | NO | NO | NO | NO | | | | NO | | | PR | | | SD | | |  |  |  |  |
| YES | | | YES | NO | YES | NO | | | | NO | | | NR | | | SD | | |  |  |  |  |
| NO | | | NO | NO | YES | NO | | | | NO | | | NR | | | SD | | |  |  |  |  |
| Year | | Reference | | | | | Gender | | Age at onset | | | Age at diagnosis | | | Bone involvement | | | Visceral involvement | | | Symptoms | |
| 2020 | | Schneider et al., 2020. Acta Orthopaedica;91(2): 209-214. | | | | | M | | 16 | | | 21 | | | SP, UL, LL | | | NR | | | Pain, swelling | |
| 2020 | | Schneider et al., 2020. Acta Orthopaedica;91(2): 209-214. | | | | | M | | 10 | | | 13 | | | SP, RI, UL, LL | | | NR | | | Pain, CSFL, chylothorax | |
| 2020 | | Schneider et al., 2020. Acta Orthopaedica;91(2): 209-214. | | | | | F | | 11 | | | 11 | | | LL | | | NR | | | Pain, swelling | |
| 2020 | | Schneider et al., 2020. Acta Orthopaedica;91(2): 209-214. | | | | | M | | 39 | | | 42 | | | UL | | | NR | | | Pain | |
| 2020 | | Schneider et al., 2020. Acta Orthopaedica;91(2): 209-214. | | | | | M | | 10 | | | 12 | | | SP | | | NR | | | Pain | |
| 2020 | | Stephens et al., 2020. Journal of Clinical Neuroscience;72: 486-492. | | | | | M | | 5 | | | 8 | | | CR, SP, UL, LL | | | Spleen | | | Chiari I malformation, NSI, meningitis | |
| 2020 | | Vaishya et al., 2020. Journal of Orthopaedics;18: 177-180. | | | | | F | | 68 | | | 68 | | | LL | | | NR | | | Pain, pathological fracture | |
| 2020 | | Yokoi et al., 2020. Surgical Neurology International;11. | | | | | F | | 13 | | | 14 | | | SP, RI | | | NR | | | Pain, NSI, CSFL, PE | |
| 2020 | | Zanelli et al., 2020. International Journal of Surgical Pathology;28(1): 76-77. | | | | | F | | NR | | | 49 | | | SP, RI | | | NR | | | Pain | |
| 2021 | | Duczkowski et al., 2021. Polish journal of pathology; 72(2): 174-179. | | | | | M | | 8 | | | 8 | | | UL | | | NR | | | Pain | |
| 2021 | | Esper et al., 2021. Oxford Medical Case Reports;2021(1): 8-12. | | | | | F | | 60 | | | 60 | | | LL | | | NR | | | Pain, swelling | |
| 2021 | | Evsyukov et al., 2021. British Journal of Neurosurgery. | | | | | M | | 54 | | | 58 | | | CR, SP | | | NR | | | Pain, pathological fracture | |
| Surgery | | | Sirolimus | Interferon | Bisphosphonates | RT | | | | PT | | | Quality of life | | | Imaging evaluation | | |  |  |  |  |
| NO | | | NO | NO | YES | NO | | | | NO | | | NR | | | NR | | |  |  |  |  |
| YES | | | YES | YES | YES | NO | | | | NO | | | NR | | | SD | | |  |  |  |  |
| YES | | | NO | NO | YES | NO | | | | NO | | | NR | | | NR | | |  |  |  |  |
| NO | | | NO | NO | YES | NO | | | | NO | | | NR | | | SD | | |  |  |  |  |
| YES | | | NO | NO | YES | NO | | | | NO | | | NR | | | SD | | |  |  |  |  |
| YES | | | YES | NO | YES | NO | | | | NO | | | PR | | | PD | | |  |  |  |  |
| YES | | | NO | NO | NO | NO | | | | NO | | | PR | | | SD | | |  |  |  |  |
| YES | | | YES | NO | YES | NO | | | | NO | | | PR | | | NR | | |  |  |  |  |
| NR | | | NR | NR | NR | NR | | | | NR | | | NR | | | NR | | |  |  |  |  |
| NR | | | NR | NR | NR | NR | | | | NR | | | NR | | | NR | | |  |  |  |  |
| NO | | | NO | NO | YES | NO | | | | YES | | | PR | | | NR | | |  |  |  |  |
| YES | | | NO | NO | NO | NO | | | | YES | | | PR | | | PD | | |  |  |  |  |
| Year | | Reference | | | | | Gender | | Age at onset | | | Age at diagnosis | | | Bone involvement | | | Visceral involvement | | | Symptoms | |
| 2021 | | Grönroos, M. and A. Palomäki, 2021. Journal of Medical Case Reports;15(1). | | | | | M | | NR | | | 23 | | | SP, RI, LL | | | NR | | | Spinal deformity, dyspnea, NSI, PE | |
| 2021 | | Kamble et al., 2021. J Orthop Case Rep;11(1): 101-103. | | | | | M | | NR | | | 22 | | | UL | | | NR | | | Pathological fracture | |
| 2021 | | López García et al., 2021. Reumatologia Clinica;17(8): 485-488. | | | | | M | | 14 | | | 20 | | | CR, SP, RI, LL | | | Spleen | | | Pain, spinal deformity | |
| 2021 | | Momanu et al., 2021. Medicina;57(7). | | | | | F | | NR | | | 18 | | | SP, RI, UL, LL | | | Spleen | | | Pain, pathological fracture, dyspnea, NSI, chylothorax | |
| 2021 | | Rana et al., 2021. Lymphology;54(4): 182-194. | | | | | F | | 3 | | | 25 | | | LL | | | NR | | | Pain, swelling | |
| 2021 | | Rana et al., 2021. Lymphology;54(4): 182-194. | | | | | M | | 2 | | | 4 | | | LL | | | NR | | | Pain, pathological fracture | |
| 2021 | | Rana et al., 2021. Lymphology;54(4): 182-194. | | | | | M | | 6 | | | 6 | | | SP, RI, UL | | | NR | | | Swelling, chylothorax | |
| 2021 | | Rana et al., 2021. Lymphology;54(4): 182-194. | | | | | M | | 0 | | | 0.25 | | | CR | | | NR | | | Swelling | |
| 2021 | | Rana et al., 2021. Lymphology;54(4): 182-194. | | | | | M | | 0 | | | 13 | | | CR, SP, RI, UL, LL | | | NR | | | Pain | |
| 2021 | | Rana et al., 2021. Lymphology;54(4): 182-194. | | | | | M | | 0.33 | | | 0.42 | | | SP, UL, LL | | | NR | | | Dyspnea, chylothorax | |
| 2021 | | Rana et al., 2021. Lymphology;54(4): 182-194. | | | | | M | | 0 | | | 6 | | | CR | | | NR | | | Meningitis, unilateral deafness | |
| 2021 | | Rana et al., 2021. Lymphology;54(4): 182-194. | | | | | M | | 0 | | | 14 | | | CR | | | NR | | | Meningitis | |
| Surgery | | | Sirolimus | Interferon | Bisphosphonates | RT | | | | PT | | | Quality of life | | | Imaging evaluation | | |  |  |  |  |
| YES | | | NO | YES | YES | YES | | | | NO | | | PR | | | SD | | |  |  |  |  |
| YES | | | NO | NO | YES | NO | | | | YES | | | PR | | | SD | | |  |  |  |  |
| NO | | | YES | NO | YES | NO | | | | NO | | | NR | | | NR | | |  |  |  |  |
| YES | | | NO | NO | NO | NO | | | | NO | | | Dead | | | Dead | | |  |  |  |  |
| NO | | | YES | NO | YES | NO | | | | NO | | | PR | | | SD | | |  |  |  |  |
| NO | | | NO | NO | YES | NO | | | | NO | | | NR | | | SD | | |  |  |  |  |
| NO | | | YES | NO | YES | NO | | | | NO | | | NR | | | SD | | |  |  |  |  |
| NO | | | NO | YES | YES | NO | | | | YES | | | NR | | | SD | | |  |  |  |  |
| NO | | | NO | YES | YES | NO | | | | NO | | | PR | | | SD | | |  |  |  |  |
| NO | | | YES | NO | YES | NO | | | | NO | | | NR | | | SD | | |  |  |  |  |
| NO | | | YES | NO | YES | NO | | | | NO | | | NR | | | NR | | |  |  |  |  |
| YES | | | NO | NO | NO | NO | | | | NO | | | PR | | | SD | | |  |  |  |  |
| Year | | Reference | | | | | Gender | | Age at onset | | | Age at diagnosis | | | Bone involvement | | | Visceral involvement | | | Symptoms | |
| 2021 | | Rana et al., 2021. Lymphology;54(4): 182-194. | | | | | F | | 0 | | | 0.17 | | | CR | | | NR | | | Swelling | |
| 2021 | | Saify et al., 2021. J Oral Maxillofac Pathol;25(Suppl 1): S7-s10. | | | | | F | | NR | | | NR | | | CR | | | NR | | | Pain | |
| 2021 | | Saldarriaga et al., 2021. European Journal of Case Reports in Internal Medicine;8(8). | | | | | M | | NR | | | NR | | | CR | | | NR | | | Pain | |
| 2021 | | Thompson, A. A. and S. Patrawala, 2021. BMJ Case Reports;14(1). | | | | | F | | 60 | | | NR | | | CR, SP, S, UL | | | NR | | | Pain, pathological fracture, dyspnea, NSI, chylothorax | |
| 2021 | | Toga et al., 2021. JBJS Case Connector;11(1). | | | | | F | | 13 | | | 13 | | | RI, SP | | | NR | | | Pain, spinal deformity | |
| 2021 | | Watanabe et al., 2021. Yonago Acta Medica;64(3): 318-323. | | | | | M | | 13 | | | 16 | | | CR | | | NR | | | Pain, CSFL, meningitis | |
| 2022 | | Ahmetgjekaj et al., 2022. Radiology Case Reports;17(9): 3243-3246. | | | | | M | | 39 | | | 41 | | | SP, RI, S, UL | | | NR | | | Pain, NSI | |
| 2022 | | Anthony et al., 2022. Lymphatic Research and Biology;20(2): 118-124. | | | | | F | | 1 | | | 1 | | | CR, SP | | | NR | | | Meningitis, hearing loss | |
| 2022 | | Aouad et al., 2022. Child's Nervous System;38(2): 455-460. | | | | | F | | NR | | | 3 | | | CR | | | NR | | | Swelling, Chiari I malformation, CSFL | |
| 2022 | | Hong et al., 2022. Medicine;101(50): E32105. | | | | | M | | 67 | | | 67 | | | SP, RI, UL, LL | | | NR | | | Chylothorax | |
| 2022 | | Hyseni et al., 2022. Radiology Case Reports;17(3): 492-495. | | | | | M | | 50 | | | 53 | | | CR, SP, RI, S, UL, LL | | | NR | | | Pain, NSI, | |
| 2022 | | Iyengar et al., 2022. J Clin Immunol;42(3): 706-708. | | | | | F | | 0.42 | | | 0.42 | | | SP, RI, UL | | | NR | | | Swelling, dyspnea, PE | |
| Surgery | | | Sirolimus | Interferon | Bisphosphonates | RT | | | | PT | | | Quality of life | | | Imaging evaluation | | |  |  |  |  |
| NO | | | NO | NO | NO | NO | | | | NO | | | NR | | | SD | | |  |  |  |  |
| NR | | | NR | NR | NR | NR | | | | NR | | | NR | | | NR | | |  |  |  |  |
| NO | | | YES | NO | YES | NO | | | | NO | | | PR | | | SD | | |  |  |  |  |
| YES | | | YES | NO | NO | NO | | | | NO | | | Dead | | | Dead | | |  |  |  |  |
| YES | | | NO | YES | YES | NO | | | | NO | | | PR | | | SD | | |  |  |  |  |
| YES | | | NO | NO | YES | NO | | | | NO | | | PR | | | SD | | |  |  |  |  |
| YES | | | NO | NO | YES | NO | | | | NO | | | NR | | | NR | | |  |  |  |  |
| NO | | | YES | NO | YES | NO | | | | NO | | | PR | | | SD | | |  |  |  |  |
| YES | | | NO | NO | NO | NO | | | | NO | | | PR | | | PR | | |  |  |  |  |
| NO | | | NO | NO | YES | NO | | | | NO | | | PR | | | NR | | |  |  |  |  |
| NO | | | NO | NO | YES | NO | | | | NO | | | NR | | | NR | | |  |  |  |  |
| NO | | | YES | NO | NO | NO | | | | NO | | | PR | | | NR | | |  |  |  |  |
| Year | | Reference | | | | | Gender | | Age at onset | | | Age at diagnosis | | | Bone involvement | | | Visceral involvement | | | Symptoms | |
| 2022 | | Krishnan et al., 2022. Surgical Neurology International;13. | | | | | F | | 12 | | | 12 | | | SP | | | NR | | | Pain, spinal deformity | |
| 2022 | | Maroufi et al., 2022. Childs Nervous System;38(4): 695-703. | | | | | M | | 10 | | | 11 | | | CR, SP | | | NR | | | Pain, spinal deformity | |
| 2022 | | Mbaga et al., 2022. Acta Orthopaedica Belgica;88(3): 475-481. | | | | | M | | NR | | | 17 | | | UL | | | NR | | | Pathological fracture, swelling | |
| 2022 | | Mbaga et al., 2022. Acta Orthopaedica Belgica;88(3): 475-481. | | | | | M | | NR | | | 16 | | | UL | | | NR | | | Limited elbow range of motion | |
| 2022 | | Morinaga et al., 2022. NMC Case Rep J;9: 55-61. | | | | | F | | 2 | | | 33 | | | CR | | | NR | | | CSFL, meningitis | |
| 2022 | | Nomura et al., 2022. American Journal of Respiratory and Critical Care Medicine;205(11): E53-E54. | | | | | F | | NR | | | 18 | | | SP, RI | | | Lung | | | Pain, dyspnea, chylothorax | |
| 2022 | | Roy et al., 2022. Advances in Radiation Oncology;7(1). | | | | | F | | NR | | | 27 | | | CR | | | NR | | | Pathological fracture, swelling, loss of tooth | |
| 2022 | | Satta et al., 2022. BMJ Case Reports;15(11). | | | | | F | | 35 | | | 40 | | | SP, RI | | | Lung | | | Pain, dyspnea, NSI, chylothorax | |
| 2022 | | Stiefel et al., 2022. Oral Surgery Oral Medicine Oral Pathology Oral Radiology;133(2): 129-137. | | | | | F | | 59 | | | 60 | | | CR | | | NR | | | Pain, pathological fracture, swelling | |
| 2022 | | Suzuki et al., 2022. JRSM Open;13(6). | | | | | F | | NR | | | 16 | | | RI, UL | | | NR | | | Pain, pathological fracture, dyspnea, chylothorax | |
| 2022 | | Toda et al., 2022. Japanese Journal of Clinical Oncology;52(2): 197. | | | | | M | | NR | | | 46 | | | LL | | | NR | | | Pain | |
| Surgery | | | Sirolimus | Interferon | Bisphosphonates | RT | | | | PT | | | Quality of life | | | Imaging evaluation | | |  |  |  |  |
| YES | | | YES | NO | YES | NO | | | | NO | | | PR | | | SD | | |  |  |  |  |
| NO | | | NO | NO | YES | NO | | | | YES | | | PD | | | PD | | |  |  |  |  |
| YES | | | YES | NO | NO | NO | | | | NO | | | PR | | | PD | | |  |  |  |  |
| YES | | | NO | NO | NO | NO | | | | NO | | | NR | | | NR | | |  |  |  |  |
| YES | | | NO | NO | NO | NO | | | | NO | | | PR | | | NR | | |  |  |  |  |
| NR | | | NR | NR | NR | NR | | | | NR | | | NR | | | NR | | |  |  |  |  |
| YES | | | NO | NO | YES | YES | | | | NO | | | PR | | | SD | | |  |  |  |  |
| NO | | | YES | NO | YES | NO | | | | NO | | | PR | | | NR | | |  |  |  |  |
| YES | | | NO | NO | NO | NO | | | | NO | | | PR | | | NR | | |  |  |  |  |
| YES | | | YES | NO | YES | NO | | | | NO | | | PR | | | PR | | |  |  |  |  |
| NR | | | NR | NR | NR | NR | | | | NR | | | PD | | | PD | | |  |  |  |  |

| Year | Reference | Gender | Age at onset | Age at diagnosis | Bone involvement | Visceral involvement | Symptoms |
| --- | --- | --- | --- | --- | --- | --- | --- |
| 2022 | Toda et al., 2022. Japanese Journal of Clinical Oncology;52(2): 197. | M | NR | 35 | LL | NR | Pain |
| 2022 | Toivonen et al., 2022. Endocrinol Diabetes Metab Case Rep;2022. | M | 18 | 23 | SP, RI | NR | Pain, spinal deformity, dyspnea, NSI, PE |
| 2022 | Yébenes Mayordomo et al., 2022. BMC Medical Genomics;15(1). | F | NR | 45 | UL | NR | Pathological fracture |
| 2022 | Zhang et al., 2022. Front Surg;9: 981025. | M | 10 | 13 | CR, SP, RI, LL | NR | Spinal deformity, dyspnea, NSI, chylothorax |
| 2022 | Gupta et al., 2023. Pediatr Blood Cancer;70(2): e29896. | F | 6 | 6 | CR, SP, RI, UL, LL | NR | Pain, swelling |
| 2022 | Pickering et al., 2023. Joint Bone Spine;90(1): 105478. | F | 17 | 17 | CR | NR | Pain, swelling |

| Surgery | Sirolimus | Interferon | Bisphosphonates | RT | PT | Quality of life | Imaging evaluation |
| --- | --- | --- | --- | --- | --- | --- | --- |
| NR | NR | NR | NR | NR | NR | NR | PD |
| YES | NO | YES | YES | YES | NO | PR | NR |
| NR | NR | NR | NR | NR | NR | NR | NR |
| YES | NO | NO | YES | NO | YES | PR | NR |
| NO | YES | YES | YES | NO | NO | PR | NR |
| NO | NO | NO | NO | NO | NO | PR | PR |

NR: Not Reported; M: Male; F: Female; UL: Upper Limb; LL: Lower Limb; SP: Spinal; CR: Cranial; RI: Ribs; S: Sternum;

NSI: Nervous System Impairment; PE: Pleural Effusion; CSFL: Cerebral Spinal Fluid Leakage;

RT: Radiotherapy; PT: Physical Therapy; PR: Partial Response; SD; Stable Disease; PD: Progressive Disease
